# The MCU-MECOM Axis Orchestrates Glioblastoma Progression by Remodeling Mitochondrial Dynamics and Quality Control via MAMs

**DOI:** 10.7150/ijbs.127940

**Published:** 2026-05-01

**Authors:** Xiaodong Li, Yaoliang Wang, Feifei Wu, Xin Huang, Ziwei Ni, Guangzhen Xu, Weizhong Wang, Jingjing Tie, Yunqiang Huang, Yuze Sun, Zhenhua Wang, Shujiao Li, Qianwen Zheng, Yuxuan Liu, Yousheng Wu, Chengrong Gong, Qingdong Guo, Yanling Yang, Yayun Wang

**Affiliations:** 1 Specific Lab for Mitochondrial Plasticity Underlying Nervous System Diseases, National Demonstration Center for Experimental Preclinical Medicine Education, The Fourth Military Medical University, Xi'an, 710032, China.; 2 Department of Hepatobiliary Surgery, Xi-Jing Hospital, The Fourth Military Medical University, Xi'an, 710032, China.; 3 Department of Neurosurgery, Xi-Jing Hospital, The Fourth Military Medical University, Xi'an, 710032, China.; 4 Department of Neurosurgery, Shaanxi Provincial People's Hospital, Xi'an, 710068, China.; 5 Department of Hepatobiliary and Pancreatic Surgery, Affiliated Hospital of Yan'an University, Yan'an, 716000, China.; 6 Department of Neurosurgery, the 960th Hospital of the PLA Joint Logistics Support Force, Jinan, Shandong 250031, China.; 7 Department of Laboratory Medicine, Da-Xing Hospital, Xi'an, 710032, China.; 8 Department of Computer Fundamentals, The Fourth Military Medical University, Xi'an, 710032, China.

**Keywords:** Glioblastoma, MCU, MAMs, MECOM, MAMs-Net

## Abstract

Glioblastoma (GBM) exhibits metabolic plasticity, relying on mitochondrial oxidative phosphorylation (OXPHOS) to support migration and therapy resistance. Although mitochondrial calcium overload typically induces apoptosis, GBM cells maintain viability under high calcium conditions. The structural and metabolic coupling mechanisms underlying this adaptation remain incompletely understood. Here, we identify a mitochondria-associated membranes (MAMs) regulatory axis driven by a positive feedback loop between the mitochondrial calcium uniporter (MCU) and the transcription factor MECOM. Using multi-omics profiling, time-resolved functional assays, and mitochondrial transfer experiments, we show that MCU-mediated calcium influx expands MAMs without triggering cell death. This influx initiates adaptive mitochondrial cristae remodeling via the Mic10/Mic60 complex and activates selective mitophagy. Pharmacological blockade and autophagy-rescue experiments (using si-ATG5 and chloroquine) indicate that this mitophagy-dependent quality control promotes tumor migration and buffers reactive oxygen species (ROS) to sustain OXPHOS capacity. Targeting the MCU-MECOM axis induces metabolic suppression and reduces glioma cell viability. To translate these findings into a diagnostic application, we developed MAMs-Net, a deep-learning framework for the automated quantification of MAMs ultrastructure from transmission electron microscope (TEM) images. In an independent external validation cohort, MAMs-Net achieved an AUC of 0.95 for glioma pathological stratification. This study characterizes an MCU-MECOM structural-metabolic circuit that supports GBM survival under calcium overload, identifying a potential therapeutic target and providing a pathophysiologically interpretable, AI-driven tool for glioma evaluation.

## Introduction

Glioblastoma (GBM) is the most common and aggressive primary malignant brain tumor in adults^1^, ^2^. Despite standard-of-care treatments, including surgical resection and chemoradiotherapy, tumor recurrence is almost inevitable^3^,^4^. This therapy resistance is closely associated with the highly invasive nature of the tumor and the presence of glioma stem cells (GSCs)^5^,^6^. Recent studies highlight metabolic reprogramming as a core hallmark of GBM plasticity. Rather than relying solely on aerobic glycolysis, aggressive GBMs and GSCs increasingly depend on mitochondrial oxidative phosphorylation (OXPHOS) and bioenergetic flexibility to support cell proliferation and migration^7^,^8^. Therefore, targeting mitochondrial homeostasis and the associated hyper-metabolic state represents a potential therapeutic strategy, though the underlying regulatory networks remain incompletely characterized.

Mitochondrial structural and functional dynamics are largely regulated by their physical interactions with the endoplasmic reticulum (ER) at mitochondria-associated membranes (MAMs)^9^,^10^. MAMs serve as signaling hubs that coordinate lipid synthesis, autophagosome formation, and intracellular calcium (Ca²⁺) shuttling^10^. Within these microdomains, the mitochondrial calcium uniporter (MCU) acts as the primary channel mediating calcium uptake. Physiological calcium influx stimulates tricarboxylic acid (TCA) cycle enzymes to facilitate ATP production; however, sustained mitochondrial calcium overload typically induces mitochondrial permeability transition pore (mPTP) opening and apoptosis^11^,^12^. Interestingly, highly malignant cancer cells, including GBM, can evade this apoptotic fate and maintain high viability under conditions of calcium hyperactivation^13^. The specific mechanisms allowing glioblastoma cells to resolve this survival paradox and exploit MCU-driven calcium signals for tumor progression require further investigation.

In this study, we investigated the ultrastructural and metabolic coupling mechanisms driven by calcium signaling in high-grade glioma (HGG). Through multi-omics and functional analyses, we identified a positive feedback loop between MCU and the transcription factor MECOM^14^,^15^. We demonstrated that MCU-mediated calcium overload in GBM triggers adaptive mitochondrial cristae remodeling via the Mic10/Mic60 complex and activates protective mitophagy to buffer reactive oxygen species (ROS), thereby sustaining high OXPHOS capacity. Using pharmacological interventions and autophagy-rescue experiments, we provided evidence that this MCU-coordinated mitophagy actively drives tumor migration. Furthermore, targeting MCU disrupted this structural-metabolic coupling, inducing a severe bioenergetic crisis that suppressed the malignant phenotype. To translate these nanoscale biological findings into potential clinical applications, we developed and externally validated MAMs-Net, a deep-learning framework for the automated identification and morphometric quantification of MAMs from transmission electron microscope (TEM) images. This approach offers a pathophysiologically interpretable method to assist in glioma stratification.

## Materials and Methods

### Bioinformatics analysis

Uniformly standardized pan-cancer data (TCGA TARGET GTEx, N = 19,131 samples, G = 60,499 genes) were retrieved from the UCSC database. For *MCU* (ENSG00000156026), expression data were extracted from samples annotated as “Solid Tissue Normal”, “Primary Solid Tumor”, “Primary Tumor”, “Normal Tissue”, “Primary Blood Derived Cancer-Bone Marrow”, and “Primary Blood Derived Cancer-Peripheral Blood”. For *MECOM* (ENSG00000085276), samples included “Primary Blood Derived Cancer-Peripheral Blood (TCGA-LAML)”, “Primary Tumor”, “Metastatic” (TCGA-SKCM), “Primary Blood Derived Cancer-Bone Marrow”, “Primary Solid Tumor”, and “Recurrent Blood Derived Cancer-Bone Marrow”. All expression values underwent log2 (x+1) transformation.

For differential expression analysis, *MCU* analysis excluded cancer types with fewer than 3 samples, retaining 34 malignancies. Differential tumor versus normal expression was evaluated using the unpaired Wilcoxon rank-sum test via the wilcox.test function implemented in the stats package of R (version 3.6.4). Similarly, differential expression analysis of *MECOM* across glioma clinical stages was performed using the same methodology in R (version 3.6.4). To account for multiple comparisons, *P*-values were adjusted using the Benjamini-Hochberg false discovery rate (FDR) method. Additionally, independent differential expression validation was performed using the GEO dataset (GSE4290).

Prognostic analysis for *MECOM* integrated TCGA prognostic data and TARGET follow-up records from the UCSC Cancer Browser. Samples with less than 30 days of follow-up or cancer types with fewer than 10 samples were excluded, yielding 44 malignancies. Cox proportional hazards regression models were constructed using the coxph function in the survival package (version 3.2-7) of R to evaluate the association between gene expression and prognosis, with statistical significance assessed via the log-rank test. The optimal cutoff value for *MECOM* gene expression was determined using the maxstat package (version 0.7-25), employing maximally selected rank statistics with multiple *P*-value approximations. Parameters constrained the minimal subgroup size to > 25% and the maximal subgroup size to < 75% of the cohort, yielding a cutoff of 1.5261. Patients were stratified by this expression threshold into high and low groups, and overall survival (OS) differences were analyzed using the survfit function from the survival package. To construct a multiparametric prognostic model, key MAMs and mitophagy-related genes were subjected to least absolute shrinkage and selection operator (LASSO)-Cox regression analysis in the CGGA cohort using the glmnet package^16^.

For pathway and interaction analyses, gene sets for cancer pathways and mitochondrial calcium transport were retrieved from the Gene Set Enrichment Analysis (GSEA) database. Functional enrichment analysis, including Gene Ontology (GO) and Kyoto Encyclopedia of Genes and Genomes (KEGG) pathways, was conducted to delineate migration and metabolism-associated programs. Venn diagram analysis comparing volcano plot-filtered differentially expressed genes (DEGs) and cancer pathway genes was performed using the Micro Bioinformatics Platform, identifying 31 overlapping genes. These intersection genes, along with the mitochondrial calcium transport gene sets, were subsequently subjected to predictive protein-protein interaction (PPI) network analysis via the STRING database. The analysis was conducted with a minimum interaction confidence score of > 0.7 and the species limited to *Homo sapiens*. Finally, to map the spatial and temporal dynamics of the MCU-MECOM axis, single-cell RNA sequencing (scRNA-seq) data of GBM were processed. Dimensionality reduction (t-SNE/UMAP) and pseudotime trajectory inference were executed using the Seurat and Monocle packages to evaluate subtype enrichment and evolutionary trajectories across tumorigenic stages^17^.

### Patient-derived brain specimens

Glioma tissue specimens (n = 82) and histologically normal human brain tissue samples (n = 6) were collected from the Department of Neurosurgery, First Affiliated Hospital of the Fourth Military Medical University between June 2023 and March 2025. Detailed clinicopathological characteristics are provided (detailed in Table S1). Additionally, an independent external validation cohort comprising 16 glioma specimens was collected from the Department of Neurosurgery, the 960th Hospital of the PLA Joint Logistics Support Force. This study received approval from the institutional ethics committee (No. KY20232288-C-1, No. KY20252229-C-1 and 2023-035) and strictly followed all relevant ethical guidelines, with written informed consent obtained from all participating patients prior to sample collection.

All glioma specimens underwent rigorous histopathological re-evaluation and molecular verification, followed by grading according to the 2021 WHO Classification of Tumors of the Central Nervous System (5th edition)^18^. The specific inclusion criteria for patients in this study were as follows: (1) newly diagnosed glioma confirmed by post-operative pathology; and (2) availability of complete baseline clinical and molecular data. The exclusion criteria were: (1) patients who had received any form of preoperative anti-tumor treatments, including radiotherapy, chemotherapy, or targeted therapy; (2) patients with a concurrent or prior history of other primary malignancies; (3) specimens with insufficient tissue volume or inadequate quality for subsequent experimental analyses.

Low-grade glioma (LGG) included Grade 1 (n = 10) and Grade 2 (n = 26). High-grade glioma (HGG) included Grade 3 (n = 24) and Grade 4 (n = 38). Control specimens (n = 6) were obtained during intracerebral hemorrhage surgeries. Neurosurgeons and neuropathologists jointly confirmed these as histologically normal brain tissues (>2 cm beyond hematoma margins), showing no macroscopic or microscopic abnormalities (including neuronal degeneration, gliosis, or microhemorrhages).

Specimens were sequentially numbered. According to size, tissues were immediately sectioned and processed as follows: (1) fixed in Gluta fixative (P1126, Solarbio, China) for MAMs-TEM; (2) snap-frozen in liquid nitrogen for MAMs-WB; (3) preserved in 4% paraformaldehyde (JR23992A, Yesen Biotechnology, Shanghai) for IF and IHC of MAMs; and (4) immersed in sterile DMEM with 1% penicillin-streptomycin for primary cell culture and calcium detection in MAMs.

### Primary cell culture

Tissues were washed thrice with pre-chilled Dulbecco's phosphate-buffered saline (DPBS) to remove hemorrhagic contaminants and necrotic debris, then minced into 1-mm³ fragments and digested in 5 mL enzymatic cocktail containing collagenase IV (1 mg/mL) and hyaluronidase (0.1 mg/mL) at 37°C with orbital shaking (150 rpm; intermittent vortexing every 10 minutes) for 45 minutes; digestion was terminated by adding equal volume of complete medium (DMEM/F12 supplemented with 10% fetal bovine serum, 1% penicillin-streptomycin, and 2 mmol/L L-glutamine), filtered through 70-μm cell strainers, and centrifuged at 1000 rpm for 5 minutes; pellets underwent erythrocyte lysis buffer treatment (5 minutes, room temperature), followed by two DPBS washes and resuspension in complete medium; cell viability (> 85%) was confirmed by trypan blue exclusion assay, after which cells were seeded at 5×10⁵ cells/mL in poly-L-lysine-coated T25 flasks and maintained at 37 °C/5% CO₂; debris was removed by 50% medium replacement at 24 hours, with full medium changes every 48 hours thereafter; at 80% confluency, cells were passaged using 0.25% trypsin-EDTA; cellular identity was validated via CD133/SOX2 immunofluorescence and short tandem repeat (STR) profiling, with experiments restricted to passages 0-5 to ensure genomic stability.

### Cell line culture

As U87MG is a typical GBM cell line, it was used for the overexpression and knockdown of MCU and MECOM, and subsequent *in vitro* experiments^19^. U87MG (CL-0238, Procell, China) was confirmed by short tandem repeat (STR) profiling; cells were cultured in Minimum essential medium (MEM) (C11095500BT, Gibco, USA) supplemented with 10% fetal bovine serum (164210-50, Procell, China) and 1% penicillin/streptomycin solution (PB180120, Procell, China), and maintained at 37°C under 5% CO₂ in a humidified incubator; all cell lines were verified mycoplasma-free.

Given that GSCs exhibit a higher preference for OXPHOS energy metabolism compared to bulk GBM cells, we selected this cell model for repeated interventions and related experiments^20^. GSCs derived from human glioblastoma specimens (ethically approved under material transfer agreements) were authenticated by CD133/SOX2 immunofluorescence and maintained in serum-free CTS™ Neurobasal Medium (A13712-01, Gibco, USA) supplemented with: 1% penicillin/streptomycin (for sterility maintenance), 2 mmol/L L-glutamine (25030081, Gibco, USA), 1x N-2 Supplement (17502048, Gibco, USA), 1% non-essential amino acids (11140050, Gibco, USA), 2% B-27™ Supplement (17504044, Gibco, USA), 20 ng/mL recombinant human bFGF (HY-P7004, MCE, USA), and 20 ng/mL EGF (HY-P7109, MCE, USA), with cultures incubated at 37 °C/5% CO₂ and routinely monitored for morphological integrity, proliferation kinetics, and medium phenol red indicators to ensure optimal growth conditions.

Normal human astrocytes (NHA, CP-H122, Procell, China) were utilized as recipient cells for the mitochondrial transfer experiments. NHA cells were cultured in Astrocyte Medium (ScienCell, USA) supplemented with 2% fetal bovine serum, astrocyte growth supplement, and 1% penicillin/streptomycin, maintained at 37 °C in a 5% CO₂ humidified incubator.

### Lentiviral-mediated overexpression and knockdown of *MCU* and *MECOM*

Lentiviral transfection services were professionally provided by Hanheng Biotechnology Co., Ltd., encompassing high-purity, endotoxin-free extraction of either target gene or short hairpin RNA (shRNA) vector plasmids alongside viral packaging helper plasmids psPAX2 and pMD2.G. Using Hanheng's proprietary Lipofiter™ transfection reagent, these three plasmids were co-transfected into 293T cells with high efficiency (detailed in Table S2). Viral supernatants were collected at 48 h and 72 h post-transfection respectively, packaged using a viral purification kit, and stored at -80 °C for subsequent applications. For lentiviral transduction, U87MG and GSCs in optimal growth phase were seeded in 6-well plates. Transfection efficiency was validated by western blot 48 h after infection, which determined an optimal multiplicity of infection (MOI) of 5 for both U87MG and GSCs. Stable transfectants were subsequently selected using 2 µg/mL puromycin, with all experimental assays conducted following a 14-day stable transfection period.

### Pharmacological treatments and siRNA transfection

To modulate MCU activity and autophagic flux, specific pharmacological agents were employed. Cells were treated with the MCU specific inhibitor MCU-i11, the MCU activator Spermine, or the autophagy inhibitor Chloroquine at optimized concentrations and durations according to experimental requirements (detailed in Table S4). For the autophagy rescue experiments, small interfering RNA (siRNA) specifically targeting human *ATG5* (*si-ATG5*) and a scrambled negative control (*si-NC*) were synthesized by Kereis (Nanjing, China). Transient transfection was performed using Lipofectamine 3000 (Invitrogen, USA) according to the manufacturer's protocol. Subsequent functional assays were conducted 48 hours post-transfection (detailed in Table S5).

### Mitochondrial isolation and transfer assay

To validate the intrinsic pathological footprint of MCU-dysregulated mitochondria, a mitochondrial transfer assay was performed. Intact mitochondria were isolated from U87MG cells (sh-MCU or control) using the Cell Mitochondria Isolation Kit (C3601, Beyotime, China) following the manufacturer's instructions. Total protein concentration of the isolated mitochondria was determined via BCA assay to ensure equal loading. Subsequently, NHA cells were co-incubated with the isolated functional mitochondria (normalized to 10 μg of mitochondrial protein per 1×10^^5^ recipient cells) in a complete medium for 24 hours to facilitate spontaneous internalization. Following extensive washing to remove uninternalized mitochondria, the recipient NHA cells were subjected to ATP production and MitoSOX detection assays.

### Transmission electron microscope and quantitative analysis

Patient-derived brain tissues and cultured U87MG and GSCs were prepared for TEM detection of 11 parameters of MAMs. They were fixed at 4 °C for 48 hours in electron microscope-grade fixative containing 2.5% glutaraldehyde in 0.1 mol/L phosphate buffer, followed by three 5-minute washes in 0.1 mol/L phosphate buffer; post-fixation was performed in 1% osmium tetroxide solution under light-protected conditions at 4 °C for 2 hours, with subsequent phosphate-buffered saline (PBS) rinses; specimens underwent graded dehydration in an ethanol series (50%, 70%, 90%; 15 minutes per concentration), transitioned in a 1:1 acetone: EPON® embedding medium mixture for 60 minutes, and finally infiltrated in pure EPON® resin overnight; the following day, samples were embedded in resin molds and polymerized at 68 °C for 48 hours; ultrathin sections (50 nm thickness) were double-stained with uranyl acetate and lead citrate (10 minutes each) prior to imaging using a JEM-1230 transmission electron microscope (JEOL Ltd., Tokyo, Japan).

TEM was heavily relied upon in this study as it represents the gold standard for resolving nanoscale organelle architectures, specifically the precise gap distances of MAMs (< 30 nm) and mitochondrial cristae morphology, which are beyond the diffraction limit of conventional light microscopy^21^, ^22^. To strictly eliminate selection bias during image acquisition, grid squares were initially selected at random under low magnification. Subsequently, at least 10 to 15 structurally intact cells per experimental group were randomly captured at high magnifications. Cells exhibiting signs of improper fixation, necrosis, or mechanical slicing damage were strictly excluded. TEM images were quantitatively analyzed via ImageJ (v1.53c, NIH, USA). To ensure rigorous objectivity, all image acquisitions and subsequent quantifications were performed independently by two experienced, blinded investigators who were unaware of the group assignments.

Total 11 MAMs-related TEM parameters were measured, including: (1) individual mitochondrial area, (2) individual mitochondrial perimeter, (3) individual mitochondrial circularity, (4) mitochondrial centroid X-coordinate, (5) mitochondrial centroid Y-coordinate, (6) mitochondrial area fraction (MAF), (7) single-cell mitochondrial density, (8) matrix-to-MRC (mitochondrial respiratory cristae) ratio, (9) MAMs-positive mitochondria, (10) vertical distance between MAMs structures, (11) contact length between MAMs structures.

### Immunofluorescence (IF) and mitochondrial network analysis (MiNA)

Tissue specimens were fixed overnight in 4% paraformaldehyde (PFA) at 4 °C, followed by dehydration in 20% sucrose solution for 48 h at 4 °C. After cryoprotection, tissues were sectioned at 30 μm thickness using a cryostat. Sections were blocked with 10% fetal bovine serum (FBS) for 30 min at room temperature, then incubated overnight at 4°C with primary antibodies against Calnexin, MCU, and MECOM, the key molecule of MAMs (Table S3) and followed by incubation with fluorescence-labeled secondary antibodies (Table S3). Nuclei were counterstained with DAPI. Confocal images were performed using a Leica STELLARIS 5 system, and image analysis was conducted in ImageJ (1.53c, NIH, USA) with consistent thresholding across all samples. Furthermore, to objectively evaluate mitochondrial dynamics, mitochondrial morphological parameters—including network branches, branch length, and connectivity—were quantitatively extracted from the fluorescent images using the Mitochondrial Network Analysis (MiNA) macro toolset implemented in ImageJ^23^. All quantitative analyses were conducted by two independent, blinded observers.

GSCs were fixed with 4% PFA and blocked. Primary antibodies of anti-CD133 and anti-SOX2 (Table S3) and fluorescence-labeled secondary antibodies (Table S3) were incubated. Nuclei were stained with DAPI. Confocal images were conducted and analyzed.

IF was employed to provide crucial spatial localization and co-expression evidence of targeted proteins at the whole-cell and tissue levels. For image acquisition, fluorescent images were captured using a confocal laser scanning microscope. To ensure quantitative reliability, at least five distinct, non-overlapping fields of view were randomly selected per slide or tissue section under identical high-magnification settings. Areas with obvious edge artifacts or tissue folding were avoided. All image acquisition parameters, including laser power, pinhole size, and exposure time, were kept strictly constant across all samples within the same experiment. The quantification of mean fluorescence intensity and protein co-localization (assessed via Pearson's correlation coefficient) was performed using ImageJ software. Similar to the TEM analysis, the IF quantification was conducted by two independent observers blinded to the experimental conditions to minimize subjective bias.

### Immunohistochemistry (IHC)

Tissue sections were deparaffinized in xylene and rehydrated through graded ethanol (100%, 95%, 80%, 60%) followed by distilled water rinses. Antigen retrieval was performed in 0.01 M sodium citrate buffer (pH 6.0) using heat-mediated epitope recovery. After endogenous peroxidase blocking with 3% H₂O₂ (10 min), sections were blocked with 3% BSA (1 h). Primary antibodies (Table S3) were applied overnight at 4 °C. Following PBS washes, HRP-conjugated secondary antibodies were incubated (30 min). DAB substrate was used for chromogenic development (5-10 min), and nuclei were counterstained with hematoxylin. Sections were dehydrated through ethanol-xylene series and mounted with neutral balsam. Quantitative analysis was performed under an Olympus CKX53 microscope.

### MAMs-calcium detection

MAMs are important bridges for calcium ion flow between ER and mitochondria^24^-^26^. Mitochondrial calcium and ER calcium in primary cells, U87MG, and GSCs were detected by using the indicator Rhod-2 AM (HY-101896, MedChemExpress, USA) and the indicator Mag-Fluo-4 AM (HY-D1498, MedChemExpress, USA) respectively (detailed in Table S4). Confocal images were conducted on a Leica system and analysis performed using ImageJ (1.53c, NIH, USA).

### Assessment of mitophagy flux

Mitophagy degree reflected MAMs structure stability. Then Mito-Keima was used to detect mitophagy in U87MG, and GSCs. Cells were transfected with Mito-Keima adenovirus (MOI: 5) for 48 hours, were then fixed with 4% PFA. Confocal images were conducted on a Leica system and analysis performed using ImageJ (1.53c, NIH, USA).

### Seahorse metabolic flux analysis (OCR and ECAR)

Cellular metabolic states, specifically the oxygen consumption rate (OCR) and extracellular acidification rate (ECAR), were comprehensively evaluated to reflect mitochondrial OXPHOS and glycolytic function, respectively. Lentivirally transduced U87MG cells and GSCs (genetically modulated for *MCU* or *MECOM*) were seeded at a density of 5×10⁴ cells/well in XF96 cell culture microplates (103794-100, Agilent, USA) and allowed to adhere overnight.

For OCR measurement, the Seahorse XF Cell Mito Stress Test Kit (103015-100, Agilent, USA) was utilized. Following equilibration in Seahorse XF Base Medium (pH 7.4) and two washes with the XF assay buffer, mitochondrial respiratory parameters were interrogated through sequential automated injections of 1.5 μM oligomycin (ATP synthase inhibitor), 1.5 μM FCCP (uncoupler), and 0.5 μM rotenone/antimycin A (Complex I/III inhibitors). OCR measurements were recorded in real-time. Key parameters derived included basal respiration, ATP production, maximal respiration, and spare respiratory capacity.

For ECAR measurement, the glycolytic function was quantified using the Seahorse XF Glycolysis Stress Test Kit according to the manufacturer's specifications. Cells were seeded at equivalent densities and sequentially treated with 10 mM glucose, 1.5 μM oligomycin, and 50 mM 2-deoxy-D-glucose (2-DG) to measure extracellular acidification kinetics.

All real-time metabolic parameters were captured via the Seahorse XFe96 Analyzer. The obtained data were subsequently normalized to the total cellular protein concentration of each well using the Seahorse Wave Desktop Software (v2.6.3.5, Agilent, USA). Key parameters derived for ECAR included glycolysis, glycolytic capacity, and glycolytic reserve.

### Mitochondrial membrane potential (MMP) detection

MMP reflected mitochondrial function state of primary cells, U87MG and GSCs. For primary cells, the cells were trypsinized using 0.25% trypsin, centrifuged at 600 g for 5 minutes at 4 °C, and resuspended in 0.5 mL culture medium containing serum or phenol red. For JC-1 staining, 1-6×10⁵ cells were incubated with 0.5 mL JC-1 working solution prepared as follows: Dilute 200× JC-1 stock solution (C2006-1, Beyotime, China) in ultrapure water at 50 μL per 8 mL, vortex thoroughly, then add 2 mL JC-1 staining buffer (5×). CCCP (C2006-4, Beyotime, China) was used as positive control. After 30-minute incubation at 37 °C, cells were washed twice with ice-cold JC-1 buffer (1×), prepared by diluting 5× JC-1 staining buffer (C2006-3, Beyotime, China) in distilled water at a 1:4 ratio. Fluorescence measurements were performed at 525 nm excitation and 590 nm emission wavelengths using a CLARIOstar® Plus microplate reader (BMG LABTECH, Germany).

For U87MG and GSCs, the cells were trypsinized (0.25%) to generate single-cell suspensions. Following centrifugation, the supernatant was discarded and cell pellets were resuspended in 500 µL TMRE staining solution (C2001S, Beyotime, China), adjusting the density to 1×10⁶ cells/mL. A positive control was established by pretreating cells with 10 µmol/L carbonyl cyanide 3-chlorophenylhydrazone (CCCP; diluted 1:1000 from 10 mmol/L kit stock). Fluorescence was analyzed using a BD FACS Celesta flow cytometer (Ex/Em = 550/575 nm), with data processed in FlowJo v10.8.1.

### Reactive oxygen species (ROS) and Mitochondrial Superoxide (MitoSOX) detection

ROS level reflected mitochondrial function state of primary cells, U87MG and GSCs, using the CellROX® Deep Red reagent (C10491, Thermo Fisher, USA). For primary cells, the cells were trypsinized with 0.25% trypsin and centrifuged (600 g, 5 min, 4°C). The pellet was resuspended in 1 mL serum-free DMEM (Gibco, USA) and cell concentration was determined. Cells were incubated with 10 μM DCFH-DA (S0033S-1, Beyotime, China) diluted 1:1000 in medium for 30 min at 37 °C, using ROS inducer (S0033S-2, Beyotime, China) as positive control. After three washes with serum-free medium to remove extracellular probe, cells were resuspended in 1 mL PBS. Fluorescence was measured at Ex/Em = 488/525 nm using CLARIOstar® Plus microplate reader (BMG LABTECH, Germany).

For U87MG and GSCs, single-cell suspensions were centrifuged (600 g, 5 min, 4 °C), followed by staining with 1 µL of the cell-permeable CellROX® Deep Red reagent. Positive and negative controls were established using tert-butyl hydroperoxide (TBHP) and N-acetyl-L-cysteine (NAC), respectively. After 30-min incubation at 37 °C, six replicates per experimental group were analyzed on a Beckman Coulter flow cytometer (Model B75442; excitation: 644 nm, emission: 665 nm). Data processing was performed using FlowJo v10.8.1.

Importantly, to specifically quantify mitochondria-derived superoxide production, the MitoSOX™ Red Mitochondrial Superoxide Indicator (M36008, Thermo Fisher, USA) was utilized. Following specific treatments, cells were washed with warm PBS and incubated with 5 μM MitoSOX reagent in the dark for 10 min at 37 °C. Nuclei were counterstained with DAPI. Fluorescent signals specific to mitochondrial ROS bursts were promptly captured via confocal microscope and quantitatively analyzed.

### Cell counting kit-8 (CCK-8) assay

The CCK-8 assay was used to evaluate the effects of lentiviral transduction targeting *MCU*/*MECOM* on the growth of U87MG and GSCs. Cells were seeded in 96-well plates at 5×10³ cells/well with 200 μL culture medium per well. Cell viability was assessed by adding 10 μL CCK-8 reagent (CA1210-500T, Solarbio, China) to each well, followed by 2 h incubation at 37°C under light-protected conditions. Absorbance was measured at 450 nm using a CLARIOstar® Plus microplate reader (BMG LABTECH, Germany), with values normalized to the 2 h baseline measurement.

### Transwell

Transwell assay was used to evaluate the effects of lentiviral transduction targeting *MCU*/*MECOM* on the migration of U87MG and GSCs. Transwell chambers (6.5 mm diameter, 8.0 μm pores; Costar 3422, USA) were used. Quantitative analysis was performed under an Olympus CKX53 microscope with migrated cells counted in five random fields per chamber using ImageJ.

### Tumorsphere formation

Tumorsphere formation was used to evaluate the effects of lentiviral transduction targeting *MCU* on the tumorigenicity of GSCs. Cells were seeded at 3×10³ cells/mL in ultra-low attachment 6-well plates (3471, Corning, USA) with horizontal agitation to ensure uniform distribution, cultured in optimized CTS Neurobasal™ Medium supplemented with growth factors and nutrient supplements. Plates were incubated at 37 °C under 5% CO₂ and saturated humidity to promote tumorsphere formation. Tumorsphere morphology was documented every 72 h. On day 10, images were captured per well using phase-contrast microscope (CKX53, Olympus, Japan). Tumorsphere diameters were measured via ImageJ employing edge detection algorithms with calibrated thresholds, and volumes were calculated as V = 4/3πr³. Formation efficiency was quantified as (tumorspheres per well/seeded cells) ×100%, while growth kinetics were assessed by diameter progression over time (n ≥ 3 independent experiments).

### Cell-derived xenograft assay in NCG mice

Male NOD/ShiLtJGpt-*Prkdc*^em26Cd52^*Il2rg*^em26Cd22^/Gpt (NCG) mice (4-6 weeks old; n = 6/group) were randomized to receive subcutaneous xenografts of MCU-modified glioma cells for studying MCU-mediated gliomagenesis. U87MG and GSCs cell suspensions (5×10⁷ cells/mL in PBS:Matrigel™, 1:1) were implanted bilaterally. Tumor growth was monitored from day 8 post-inoculation when volumes reached 50 - 100 mm³, with caliper measurements of length (L) and width (W) taken every 72 h for volume calculation (V = 0.5 × L × W²).

All procedures strictly adhered to Institutional Animal Care guidelines of the Fourth Military Medical University (No. IACUC-20231262) with predefined humane endpoints: tumor burden ≥ 10% body weight, mean diameter ≥ 16 mm, or volume > 1500 mm³. Animals exhibiting > 20% body weight loss, ulceration, infection, or necrosis were immediately euthanized via CO₂ asphyxiation. Terminal euthanasia employed graduated displacement to 100% CO₂, followed by prompt tumor dissection for molecular/histopathological analysis.

### Western blot (WB)

WB was used to analyze the expression of 21 MAM-related proteins in human brain tissues and cultured cells. Total protein was extracted and quantified using the BCA assay, followed by concentration normalization prior to western blot analysis. Target protein expression was detected with antibodies (Table S3). Membranes were incubated with secondary antibodies (Table S3) and visualized using enhanced chemiluminescence reagent (SF009, Saifise, China). Signals were captured on a Fusion FX6-XT imaging system (Vilber Lourmat, France) and quantified via ImageJ. Protein band intensities were normalized to β-Actin levels and expressed as fold-changes relative to untreated controls across three independent experiments.

### Co-immunoprecipitation (Co-IP)

Cells were harvested, washed with ice-cold PBS, and lysed in modified RIPA buffer for 30 min on ice. Lysates were centrifuged (12,000 rpm, 4 °C, 15 min), and supernatants were quantified via BCA assay with protein concentration adjusted to 2 mg/mL. 500 μg total protein was incubated overnight at 4°C with rotary agitation using 2 μg anti-MCU antibody or isotype-matched IgG control, followed by addition of pre-equilibrated Protein A/G magnetic beads (20 μL) for 4 h. Beads were washed four times with lysis buffer, and bound complexes were eluted in 1× SDS loading buffer by boiling (95°C, 10 min). Eluates were subjected to immunoblot with primary antibodies: anti-MECOM (Table S3) and anti-MCU (Table S3); secondary antibodies (Table S3) were applied prior to ECL detection.

### RNA sequencing

Total RNA was isolated from all specimens using the TIANGEN RNAprep Pure Cell/Bacteria Kit (DP430, TIANGEN Biotech, China) according to manufacturer's protocols, with RNA integrity verified by Agilent 2100 Bioanalyzer (RIN > 7.0 for all samples). Poly(A)-enriched mRNA was purified from 1 μg total RNA using NEBNext Poly(A) mRNA Magnetic Isolation Module (E7490, NEB, USA). Sequencing libraries were constructed with the NEBNext Ultra II RNA Library Prep Kit (E7770, NEB, USA) following standard fragmentation, first/second strand cDNA synthesis, end repair, adapter ligation, and PCR amplification steps. Library quality control was performed via Qubit 4.0 quantification and Agilent 2100 size distribution analysis. Paired-end sequencing (150 bp reads) was conducted on Illumina NovaSeq 6000 platforms (Annoroad Gene Technology, China) with a minimum output of 40 million raw reads per sample. Raw data quality was assessed using FastQC (v0.11.9, Babraham Bioinformatics, USA) with all samples achieving Q30 scores > 85% and Phred quality scores ≥ 30 across > 90% of bases.

### TEM-based AI prediction of glioma grade from MAMs ultrastructure

Model training and data augmentation: To predict glioma pathological grading using TEM images, we established a dual-stage artificial intelligence framework, prioritizing subcellular structure localization over morphological quantification. In the first stage, multiple instance segmentation models—including Mask R-CNN, RTMDet, MS R-CNN, and Cascade Mask R-CNN—were systematically evaluated. The Cascade Mask R-CNN architecture with a ResNet-50 backbone was ultimately selected as the organelle recognition model. This algorithm was adapted from the MMDetection open-source toolbox^27^, an extensively validated framework for object detection. To address data calibration and enhance model generalization across different TEM magnifications, we implemented robust data augmentation strategies during training. These included randomized multi-scale scaling (short edge randomly resized to 640-800 pixels, long edge 1333 pixels) and horizontal flipping (probability of 0.5). Model optimization was performed using stochastic gradient descent (momentum 0.9, weight decay 0.0001) across 200 training epochs, with an initial learning rate of 0.02, a linear warm-up for 500 iterations, and a scheduled decay by a factor of 10 at epochs 150 and 180. The effective total batch size was 16 via gradient accumulation. The internal dataset (N = 320 images) was partitioned into training and test sets at a 4:1 ratio.

Glioma grading prediction: based on the organelle segmentation results, 14 fields were defined for model construction. The WHO grade served as the prediction label. The remaining 13 input variables comprised 11 ultrastructural features (mitochondrial area, perimeter, circularity, centroid coordinates, area fraction, single-cell density, matrix-to-mitochondrial respiratory cristae ratio, MAMs-positive mitochondria, shortest vertical distance, and contact length of MAMs) and two clinical variables (patient age and sex). For each ultrastructural parameter, the mean, standard deviation, minimum, and maximum values were calculated across all mitochondrial instances within an image, constituting a 171-dimensional feature vector per image. The dataset was divided using a 7:3 stratified random split. To address class imbalance, the Synthetic Minority Over-sampling Technique (SMOTE) was applied to the training set. During classifier screening, Random Forest (RF), Support Vector Machine (SVM, RBF kernel), and Multi-Layer Perceptron (MLP, hidden layers 100-50) were compared. RF hyperparameters were optimized via five-fold cross-validation with a grid search (optimal: maximum tree depth of 10, minimum of 1 sample per leaf, minimum of 5 samples per split, class weighting 'balanced', and 200 trees).

External validation of the segmentation model: to evaluate the generalizability of the trained organelle segmentation model on TEM images from independent sources, the fully frozen Cascade Mask R-CNN was directly applied to the public Lucchi++ mitochondria segmentation dataset (165 TEM images of mouse cerebral cortex, containing mitochondria annotations)^28^. Performance metrics including mean Intersection over Union (mIoU), Dice coefficient, precision, and recall were calculated to assess its cross-domain transfer potential. Crucially, to rigorously assess the clinical generalizability of MAMs-Net, a Stage 3 independent external validation was conducted. The established diagnostic model was directly deployed to predict the grades of an external cohort of TEM images derived from the 16 independent glioma patients (Grades 1 to 4) collected from the 960th Hospital of the PLA Joint Logistics Support Force. The model's external performance and diagnostic AUC were subsequently evaluated to confirm its cross-center translational reliability.

### Statistical analysis

Statistical analyses were performed using GraphPad Prism 8. For comparison, the t-test (for parametric data) and the Mann-Whitney* U* test (for nonparametric data) were used to determine statistical significance. Where multiple comparisons were made, one-way ANOVA test (for parametric data) or Kruskal-Wallis test (for nonparametric data) were used. Results were reported as the mean ± standard deviation (SD). *P* < 0.05 was considered statistically significant.

## Results

### MCU drives calcium dysregulation and structural reinforcement of MAMs in HGG

**Figure 1:**
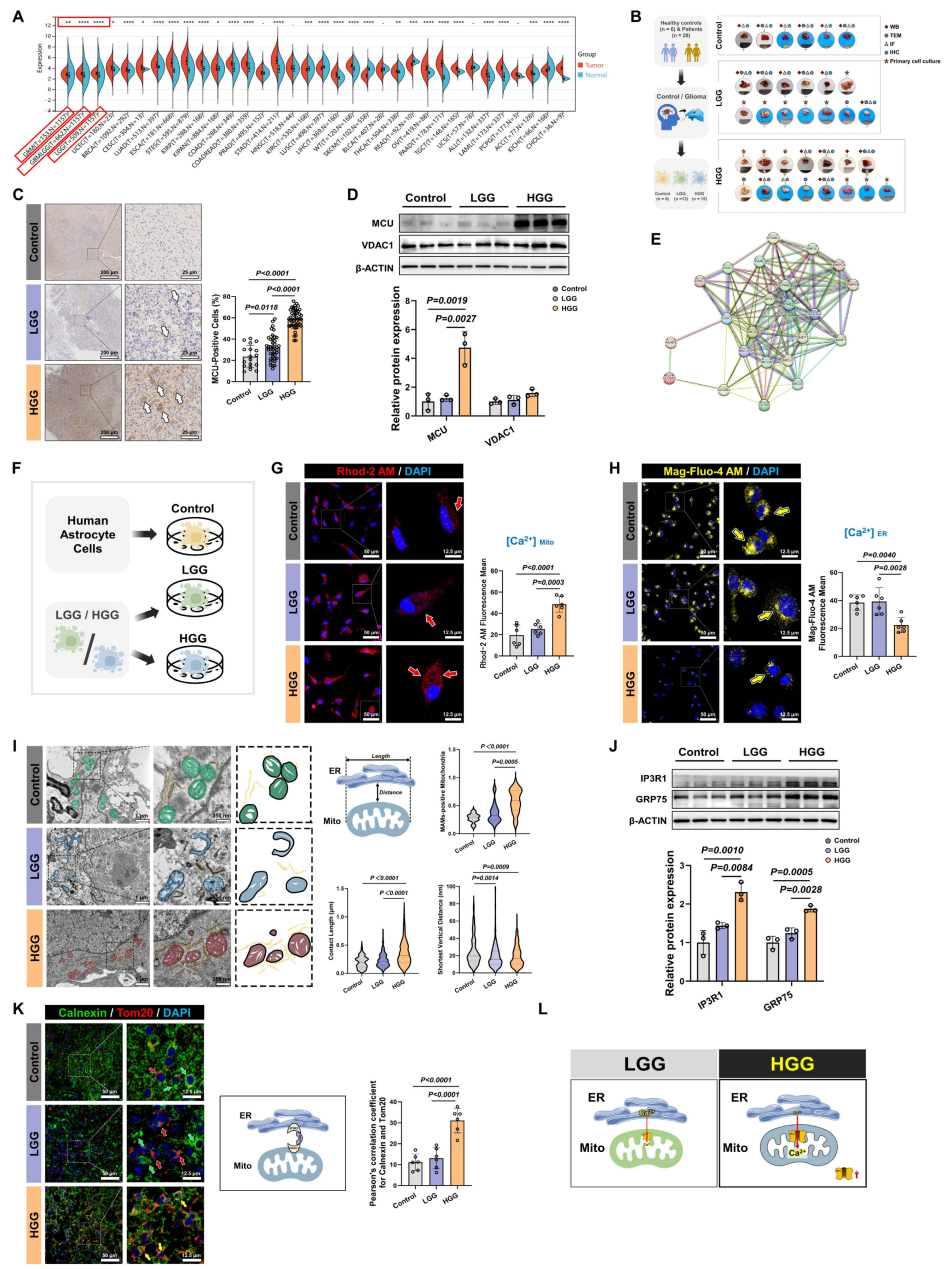
** MCU is overexpressed in high-grade glioma and promotes MAMs reinforcement and calcium dysregulation. (A)** Pan-cancer bioinformatics analysis of MCU expression levels. Violin plots display MCU mRNA levels in tumor (red) versus normal (blue) tissues across various malignancies. The red boxes highlight the glioma cohorts (GBM and LGG). **(B)** Schematic illustration of the acquisition and experimental workflow for clinical specimens (Control, LGG, and HGG). **(C)** IHC images and corresponding quantitative analysis of MCU expression in Control, LGG, and HGG tissues. **(D)** Western blot analysis and relative protein quantification of MCU and VDAC1 in clinical lysates. **(E)** PPI network analysis (STRING) highlighting mitochondrial calcium transport-related molecules.** (F)** Schematic representation of primary cell cultures established from human astrocytes (Control), LGG, and HGG specimens. **(G)** Representative immunofluorescence images and quantification of mitochondrial calcium levels using the Rhod-2 AM probe in primary cultured cells. **(H)** Representative immunofluorescence images and quantification of ER calcium levels using the Mag-Fluo-4 AM probe. **(I)** Ultrastructural analysis of MAMs via TEM. Left: Representative TEM images showing mitochondria (green/blue/red) and ER (yellow) contacts. Middle: Schematic defining the quantitative MAMs parameters. Right: Quantitative analysis of MAMs-positive mitochondria (%), MAMs contact length (μm), and shortest vertical distance (nm) between ER and mitochondria. **(J)** Western blot analysis and relative protein quantification of the MAMs tethering complex proteins IP3R1 and GRP75 across clinical grades. **(K)** Representative confocal immunofluorescence images and quantitative colocalization analysis (Pearson's correlation coefficient) of Calnexin (green) and TOMM20 (red). Nuclei were counterstained with DAPI (blue). **(L)** Schematic model illustrating the progression of MAMs structural reinforcement and subsequent mitochondrial calcium overload in HGG compared to LGG. Data are presented as mean ± SD. *P < 0.05; **P < 0.01; ***P < 0.001; ****P < 0.0001.

Our comprehensive bioinformatics analysis across 33 cancer types revealed widespread dysregulation of the MCU, with the most pronounced differential expression observed in GBM and LGG (*P* = 3.5 × 10⁻²⁵) (Fig. 1A). To validate this finding, we analyzed 34 clinical specimens (6 controls, 13 LGG, 15 HGG) (Fig. 1B). IHC showed MCU levels in HGG were approximately 2-fold higher than in controls and LGG (*P* < 0.0001) (Fig. 1C). Western blot analysis further corroborated this trend, demonstrating a 5-fold increase in HGG compared to LGG, while expression of the mitochondrial outer membrane protein VDAC1 remained unchanged (Fig. 1D). PPI network analysis positioned MCU as a central hub among mitochondrial calcium transporters (Fig. 1E). Functional assessment of calcium dynamics in primary cells revealed a significant, approximately 2-fold increase in mitochondrial calcium concentration ([Ca²⁺] _Mito_) in HGG compared to LGG and controls (*P* < 0.001) (Fig. 1F and 1G). Paradoxically, endoplasmic reticulum calcium stores ([Ca²⁺] _ER_) were depleted in HGG, showing a 41.8% reduction relative to controls (*P* = 0.0040) (Fig. 1H). This aberrant calcium redistribution prompted a detailed ultrastructural analysis of MAMs. TEM quantification revealed in HGG a 10% increase in MAMs proportion (*P* < 0.0001), which was characterized by a 77.6% increase in contact length and a 20.8% reduction in the shortest vertical distance compared to controls (*P* = 0.0009) (Fig. 1I). At the molecular level, Western blot showed concurrent upregulation of key MAMs tethering components GRP75 (1.9-fold, *P* = 0.0005) and IP3R1 (2.3-fold, *P* = 0.0010) in HGG versus controls (Fig. 1J). Furthermore, Immunofluorescence co-localization of the ER marker calnexin and the mitochondrial marker TOMM20 confirmed significantly enhanced MAMs formation in HGG tissues (*P* < 0.0001) (Fig. 1K).

Collectively, these multimodal data established that HGG was characterized by MCU overexpression, enhanced MAMs connectivity, and a pathological shift in calcium storage from the ER to mitochondria (Fig. 1L).

### MCU inhibition suppresses malignant phenotypes and *in vivo* tumorigenicity in HGG

**Figure 2:**
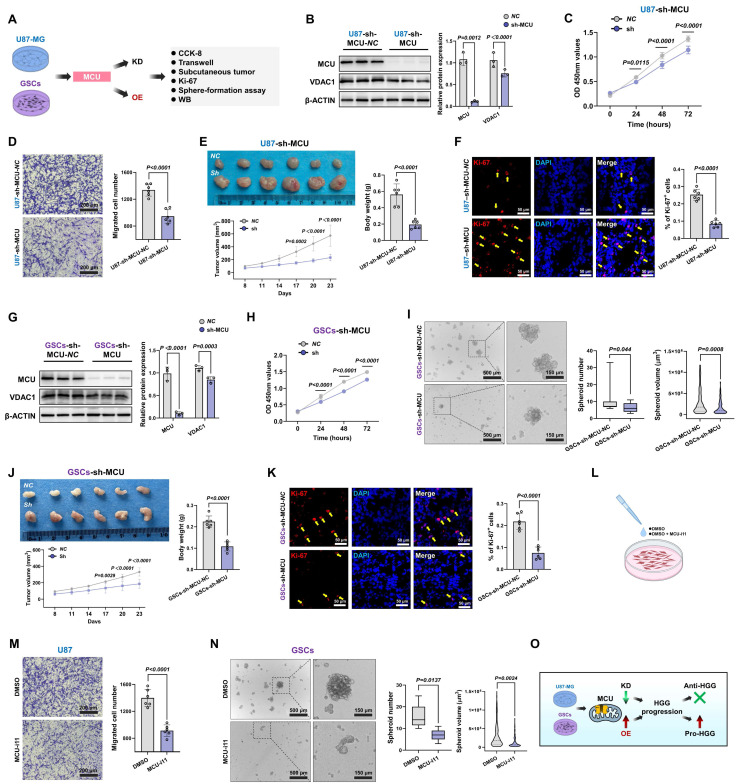
** MCU inhibition suppresses malignant phenotypes and tumorigenicity. (A)** Schematic illustration of the experimental design for lentiviral-mediated MCU knockdown (KD) and pharmacological intervention in U87MG cells and glioma stem cells (GSCs), followed by functional assays. **(B)** Western blot analysis and relative protein quantification of MCU and VDAC1 in U87MG cells following MCU knockdown (NC vs. sh-MCU). **(C)** CCK-8 assay assessing the proliferation of U87MG cells following MCU knockdown. **(D)** Representative images and quantitative analysis of Transwell migration assays for U87MG cells following MCU knockdown. **(E)** Representative images, tumor weights, and tumor growth curves of subcutaneous xenografts derived from U87MG cells following MCU knockdown. **(F)** Representative confocal immunofluorescence images and quantitative analysis of Ki-67 (red) expression in U87MG xenograft tissues. Nuclei were counterstained with DAPI (blue). **(G)** Western blot analysis and relative protein quantification of MCU and VDAC1 in GSCs following MCU knockdown. **(H)** CCK-8 proliferation assay of GSCs after MCU knockdown. **(I)** Representative images and statistical analysis of tumorsphere numbers and volumes formed by GSCs following MCU knockdown. **(J)** Representative images, tumor weights, and tumor growth curves of subcutaneous xenografts derived from GSCs following MCU knockdown. **(K)** Representative confocal immunofluorescence images and quantitative analysis of Ki-67 (red) expression in GSCs xenograft tissues. **(L)** Schematic representation of the pharmacological intervention using the specific MCU inhibitor, MCU-i11. **(M)** Representative images and quantitative analysis of Transwell migration assays for U87MG cells treated with DMSO or MCU-i11. **(N)** Representative images and statistical analysis of tumorsphere formation by GSCs treated with DMSO or MCU-i11. **(O)** Schematic summary illustrating that MCU acts as a pro-tumorigenic factor in HGG progression, while its genetic knockdown or pharmacological inhibition exerts potent anti-tumor effects. Data are presented as mean ± SD.

Having elucidated the close association between calcium dyshomeostasis within MAMs structure and MCU, we further investigated the functional impact of MCU on glioma cell fate determination and therapeutic sensitivity. Given the pivotal involvement of MCU in glioma calcium homeostasis, we systematically evaluated the modulation of malignant phenotypes following MCU targeting in HGG (Fig. 2A). Lentiviral shRNA was used to establish U87MG cell models with graded MCU knockdown (sh-MCU). Western blot analysis confirmed effective MCU knockdown alongside a significant reduction in VDAC1 expression (Fig. 2B). Functional assays demonstrated that sh-MCU significantly inhibited cellular proliferation, with CCK-8 assays showing a 16.5% decrease in viability at 72 hours (*P* < 0.0001), and reduced migration capacity, as Transwell assays evidenced by 29.3% fewer transmigrated cells (*P* < 0.0001) (Fig. 2C and 2D). In NCG mouse subcutaneous xenograft models, sh-MCU treatment reduced tumor volume by 59.3% and weight by 65.7% (*P* < 0.0001), accompanied by significantly decreased Ki-67 positivity (*P* < 0.0001) (Fig. 2E and 2F). Conversely, MCU overexpression (OE-MCU) enhanced proliferative and migratory capacities in U87MG cells, confirming the tumor-suppressive efficacy of MCU inhibition in vivo (Fig. S1A-C).

Given the critical role of GSCs in tumor recurrence and chemoresistance, we further dissected MCU function within this subpopulation^6^, ^29^. We identified GSCs through CD133/SOX2 dual-positivity (Fig. S1D). Subsequent functional analysis demonstrated that sh-MCU suppressed mitochondrial calcium uniporter expression, reduced cellular proliferation by 71% at 72 hours in CCK-8 assays (*P* < 0.0001), and impaired self-renewal capacity, with tumorsphere formation assays showing 33.6% fewer tumorspheres accompanied by 43.7% volume reduction (Fig. 2G-I). In xenograft models, sh-MCU reduced GSCs-derived tumor volume by 43.8% and weight by 51.7% (*P* < 0.0001), with Ki-67 positivity decreasing to less than 50% of the control levels (*P* < 0.0001) (Fig. 2J and 2K). Conversely, OE-MCU upregulated calcium uptake molecules and enhanced malignant phenotypes in GSCs, demonstrating bidirectional regulatory plasticity of MCU in GSCs (Fig. S1E-G).

Importantly, to validate the target specificity and therapeutic potential of MCU, we introduced the specific pharmacological inhibitor MCU-i11 (Fig. 2L). Consistent with the genetic knockdown models, pharmacological inhibition of MCU profoundly suppressed the migratory capacity of U87MG cells and disrupted the self-renewal tumorsphere formation of GSCs (Fig. 2M and 2N).

This study demonstrated that targeted genetic and pharmacological inhibition of MCU simultaneously suppressed the proliferation, migration, and regeneration capabilities of both bulk tumor cells and stem-like subpopulations, in a manner significantly correlated with MCU expression levels (Fig. 2O). Particularly significant was MCU's regulation of stemness features in GSCs, suggesting its potential role in promoting therapy resistance through reprogramming the tumor metabolic microenvironment, thereby providing a theoretical foundation for developing combination therapeutic strategies based on calcium homeostasis modulation.

### MCU reduction impairs MRC remodeling through disruption of MAMs-dependent calcium shuttling

**Figure 3:**
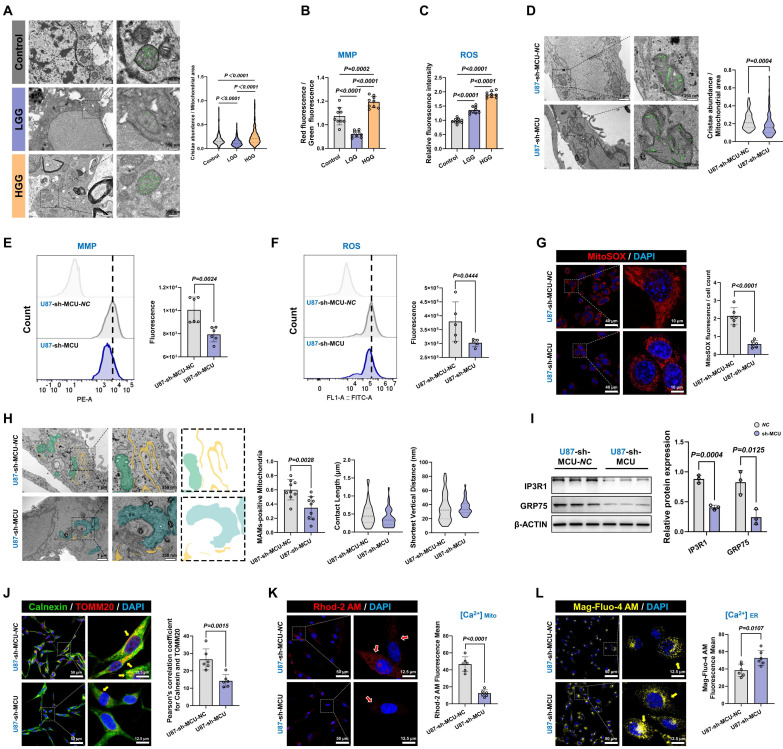
** MCU disruption impairs MRC remodeling through MAMs-dependent calcium shuttling. (A)** Representative TEM images and quantitative analysis of MRC abundance (highlighted in green) across clinical grades. Analyzed mitochondria numbers: Control (n = 390), LGG (n = 544), HGG (n = 546). **(B)** Quantitative analysis of MMP in clinical specimens using JC-1 staining (ratio of red to green fluorescence). **(C)** Quantitative analysis of global ROS levels in clinical specimens. **(D)** Representative TEM images and quantitative analysis of MRC abundance (green) in U87MG cells following MCU knockdown. Analyzed mitochondria numbers: sh-MCU-NC (n = 108), sh-MCU (n = 115). **(E)** Flow cytometry analysis and quantification of MMP in U87MG cells following MCU knockdown. **(F)** Flow cytometry analysis and quantification of intracellular ROS levels in U87MG cells following MCU knockdown. **(G)** Representative confocal images and quantitative analysis of mitochondria-specific superoxide production using the MitoSOX probe (red) in U87MG cells. Nuclei were counterstained with DAPI (blue). **(H)** Ultrastructural analysis of MAMs in U87MG cells via TEM. Left: Representative images showing mitochondria (green/blue) and ER (yellow) contacts. Right: Quantitative analysis of MAMs-positive mitochondria, contact length, and shortest vertical distance. **(I)** Western blot analysis and relative protein quantification of MAMs tethering proteins IP3R1 and GRP75 in U87MG cells following MCU knockdown. **(J)** Representative confocal immunofluorescence images and quantitative colocalization analysis (Pearson's correlation coefficient) of Calnexin (green) and TOMM20 (red). Nuclei were counterstained with DAPI (blue). **(K)** Representative confocal images and quantification of mitochondrial calcium levels using the Rhod-2 AM probe (red) in U87MG cells. **(L)** Representative confocal images and quantification of ER calcium levels using the Mag-Fluo-4 AM probe (yellow) in U87MG cells. Data are presented as mean ± SD.

Prior studies have established that hyperactivated MRC potentiated the invasive capacity of malignant tumors^30^, ^31^. Our ultrastructural analysis of clinical glioma specimens revealed that HGG progression is associated with distinct mitochondrial abnormalities. TEM quantification demonstrated a 34.2% elevation in MRC abundance per mitochondrial area compared to LGG (*P* < 0.0001) (Fig. 3A). These structural changes were accompanied by MMP hyperpolarization (*P* = 0.0002) and a 1.9-fold increase in ROS levels compared to controls (*P* < 0.0001) (Fig. 3B and 3C). Systematic interrogation of MCU regulation in U87MG showed that sh-MCU triggered cristae collapse, as evidenced by a 22.3% reduction in MRC abundance (Fig. 3D). This structural defect was accompanied by a 21.1% decrease in MMP and a significant reduction in ROS levels (Fig. 3E and 3F). Importantly, to specifically delineate the source of oxidative stress, we employed the MitoSOX probe, which confirmed a profound reduction in mitochondria-specific superoxide production following MCU knockdown (*P* < 0.0001) (Fig. 3G). Further ultrastructural evaluation via TEM confirmed 42.2% fewer MAMs contact sites and altered gap distances (*P* = 0.0028) (Fig. 3H). Mechanistic investigations demonstrated that sh-MCU significantly downregulated MAMs-tethering proteins GRP75 (*P* = 0.0125) and IP3R1 (*P* = 0.0004) (Fig. 3I). Consistent with these molecular changes, confocal imaging showed 46.6% reduced MAMs co-localization between TOMM20 and calnexin (Fig. 3J). Consequently, this structural uncoupling severely disrupted local calcium shuttling; calcium probes revealed a concomitant 73.3% mitochondrial calcium store depletion (P < 0.0001) and a 1.4-fold ER calcium retention (Fig. 3K and 3L), indicating that MCU's transmembrane calcium flux was closely related to mitochondrial quality control and redox homeostasis.

To validate this mechanism's universal applicability in tumor stemness maintenance, we replicated the experimental paradigm in GSCs (Fig. S1H-P). sh-MCU treatment triggered a 45.1% reduction in MRC abundance, concurrent suppression of the MMP and mitochondrial-specific ROS axis, mitochondrial calcium store depletion, 1.4-fold ER calcium retention, and GRP75 downregulation. Confocal imaging revealed 43.5% diminished MAMs co-localization, and TEM quantification confirming 38.5% fewer MAMs contact sites. Conversely, MCU overexpression (OE-MCU) elicited opposite phenotypes across all measured parameters (Fig. S2 and Fig. S3).

Collectively, these results demonstrated that MCU overexpression promoted calcium transfer from the ER to mitochondria, establishing a pro-tumorigenic ER-mitochondrial coupling microenvironment characterized by enhanced MMP and ROS signaling. The MCU-MAMs-cristae axis thus represented a core metabolic switch for glioma stemness sustenance.

### Targeting the MCU-MAMs axis impaired mitochondrial fission and mitophagy, thereby preventing metabolic reprogramming in HGG

**Figure 4:**
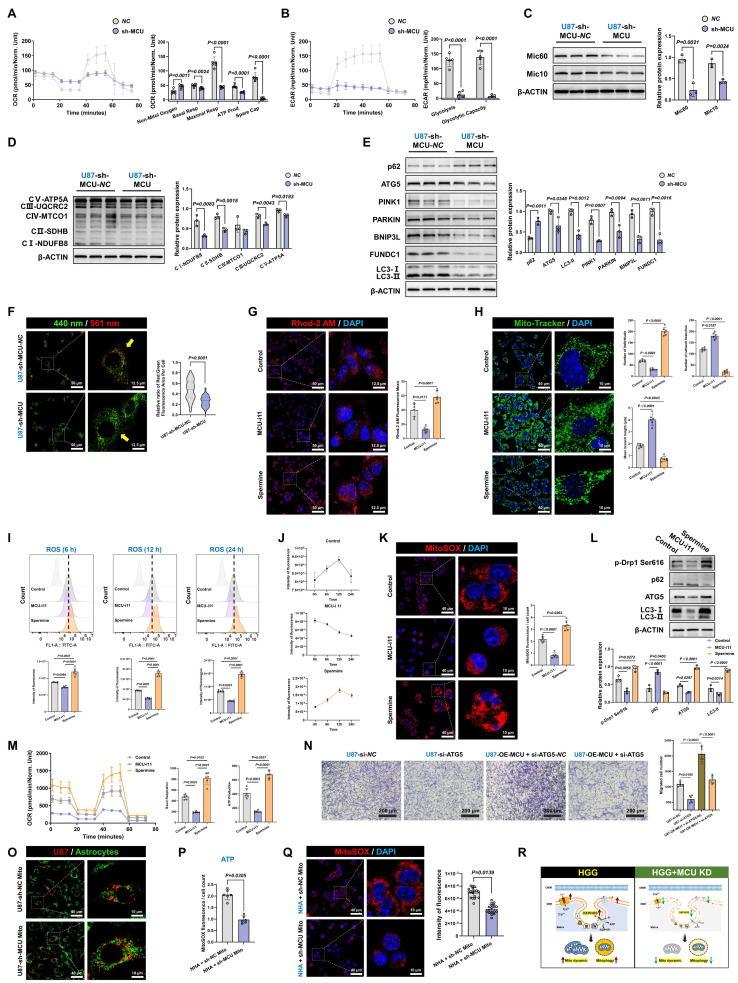
** Targeting MCU disrupts the mitochondrial fission-mitophagy axis and metabolic reprogramming in glioma. (A)** OCR analysis evaluating mitochondrial respiration in U87MG cells following MCU knockdown. **(B)** ECAR analysis assessing glycolytic function in U87MG cells following MCU knockdown. **(C)** Western blot analysis and relative protein quantification of the MICOS complex subunits Mic60 and Mic10 following MCU knockdown. **(D)** Western blot analysis of OXPHOS complex subunits (CI-CV) in U87MG cells following MCU knockdown. **(E)** Western blot analysis and quantification of key autophagy and mitophagy-related proteins (p62, ATG5, PINK1, PARKIN, BNIP3L, FUNDC1, and LC3-I/II) following MCU knockdown. **(F)** Representative confocal images and quantitative analysis of mitophagy flux using the Mito-Keima probe in U87MG cells (excitation at 440 nm for neutral pH and 561 nm for acidic pH). **(G)** Representative confocal images and quantification of mitochondrial calcium levels using Rhod-2 AM (red) following pharmacological intervention with the MCU-i11 or Spermine. **(H)** Mitochondrial network morphological analysis using Mito-Tracker Green. Right panels show the quantitative extraction of network parameters (number of individuals, network branches, and mean branch length) via the MiNA toolset. **(I** and** J)** Time-resolved flow cytometry analysis **(I)** and corresponding quantification trends **(J)** of intracellular ROS levels at 6h, 12h, and 24 h post-treatment with MCU-i11 or Spermine. **(K)** Representative confocal images and quantitative analysis of mitochondria-specific superoxide production using MitoSOX (red) under pharmacological intervention. Nuclei were counterstained with DAPI (blue). **(L)** Western blot analysis of mitochondrial fission (p-Drp1 Ser616) and autophagy markers under pharmacological modulation of MCU. **(M)** OCR analysis demonstrating metabolic shifts following treatment with MCU-i11 or Spermine. **(N)** Representative images and quantitative analysis of Transwell migration assays illustrating the causal rescue of MCU-driven migration by si-ATG5. **(O)** Representative images of the mitochondrial transfer assay, demonstrating the internalization of isolated functional mitochondria (from sh-NC or sh-MCU U87MG cells) into NHA. **(P)** Quantification of ATP production in recipient NHA cells following mitochondrial transfer. **(Q)** Representative confocal images and quantification of MitoSOX (red) in recipient NHA cells following mitochondrial transfer. **(R)** Schematic diagram illustrating the therapeutic mechanism: targeting MCU induces calcium depletion, blocks mitophagy, and triggers a severe bioenergetic crisis. Data are presented as mean ± SD.

Given the intimate association between mitochondrial OXPHOS and drug resistance in glioma, we explored whether targeting the MCU-MAMs axis could potentiate conventional therapies by remodeling metabolic vulnerability. In U87MG, sh-MCU reduced mitochondrial basal respiration by 20.4% and ATP synthesis by 44.3%, indicating MCU ablation triggered a bioenergetic crisis (Fig. 4A). Accompanying this suppression of OXPHOS, the characteristic compensatory hyper-glycolysis seen in HGG was also attenuated, with ECAR measurements showing a 90.8% reduction in glycolytic activity (Fig. 4B). Mechanistically, this metabolic collapse was associated with reduced expression of the mitochondrial cristae remodeling regulators Mic60 and Mic10, and a subsequent downregulation of multiple OXPHOS complex subunits (CI-CV) (Fig. 4C and 4D). Furthermore, sh-MCU significantly suppressed the antioxidant markers superoxide dismutase 1 (SOD1) and glutathione peroxidase 4 (GPX4) (Fig. S4A). This structural-functional disruption triggered cascading autophagic dysregulation. Western blot analysis revealed that autophagic flux was inhibited, as evidenced by significant reductions in autophagy-related protein 5 (ATG5) and microtubule-associated protein 1 light chain 3-II (LC3-II), alongside sequestosome-1 (p62) accumulation. Concurrently, both the ubiquitin-dependent mitophagy pathway, involving PTEN-induced putative kinase 1 (PINK1) and Parkin RBR E3 ubiquitin-protein ligase (PARKIN), and the receptor-mediated pathway, involving BCL2 interacting protein 3 like (BNIP3L) and FUN14 domain containing 1 (FUNDC1), were profoundly suppressed (Fig. 4E). Mito-Keima assays corroborated these results, showing a significant decrease in mitophagy signals (Fig. 4F). Finally, further assessment of the dynamic characteristics and morphological structure of mitochondria indicated a predominant shift toward mitochondrial fusion (Fig. S4B and S4C).

To further validate target specificity and delineate the temporal dynamics of this metabolic shift, we employed the specific pharmacological MCU inhibitor, MCU-i11, and the activator, Spermine. Pharmacological modulation effectively mirrored the genetic models, with MCU-i11 severely depleting mitochondrial calcium (Fig. 4G). Importantly, Mitochondrial Network Analysis (MiNA) quantitatively demonstrated that MCU-i11 significantly reduced mitochondrial branch lengths and network complexity (Fig. 4H). Importantly, time-resolved functional measurements revealed a dynamic state transition in ROS production. Tracking ROS levels at 6, 12, and 24 hours demonstrated that MCU inhibition progressively suppressed oxidative stress, while Spermine induced a sustained ROS burst, which was further confirmed to be mitochondria-specific using the MitoSOX probe (Fig. 4I-K). These temporal redox changes were accompanied by corresponding shifts in fission (p-Drp1 Ser616) and autophagic flux markers, ultimately culminating in a robust suppression of OCR by MCU-i11 (Fig. 4L-M). Importantly, these mechanistic findings were universally reproducible in GSCs models (Fig. S4D-L). Conversely, MCU overexpression enhanced mitochondrial homeostasis and reinforced metabolic adaptability in both U87MG and GSCs through increased fission, mitophagy activation, and metabolic reprogramming (Fig. S5 and S6A-I).

A critical question remained as to whether the observed autophagic activation merely accompanied these metabolic shifts or actively drove malignant progression. To address this, we performed functional rescue experiments by specifically silencing ATG5 (si-ATG5) in MCU-overexpressing cells. Western blot analysis confirmed that ATG5 knockdown effectively blocked the MCU-driven autophagic flux, evidenced by the targeted reduction of ATG5 and LC3-II, and the concomitant accumulation of p62 (Fig. S6J). Notably, the disruption of autophagy significantly impaired cell migration and completely abrogated the enhanced migratory capacity induced by MCU overexpression (Fig. 4N). In parallel at the molecular level, ATG5 silencing successfully reversed the MCU-induced upregulation of epithelial-mesenchymal transition (EMT) markers (N-Cadherin and Vimentin) and glioma stemness markers (CD133 and SOX2) (Fig. S6K). Together, these findings provided direct causal evidence that autophagy actively supports tumor invasiveness and stemness maintenance, acting as an essential downstream effector rather than a mere bystander in MCU-driven glioma progression.

Furthermore, to firmly establish MCU as the central regulator of mitochondrial quality, we transferred mitochondria isolated from sh-MCU-treated U87MG cells into normal human astrocytes (NHA) (Fig. 4O). NHA cells receiving MCU-deficient mitochondria exhibited significantly reduced ATP production and impaired mitochondrial ROS buffering compared to those receiving control mitochondria, underscoring the intrinsic pathological footprint of MCU-dysregulated mitochondria (Fig. 4P and 4Q).

Taken together, these data supported a model in which MCU targeting disrupted glioma bioenergetics through coordinated effects on metabolic, structural, and autophagic pathways (Fig. 4R). This triple-axis impairment led to suppressed energy production, disrupted mitochondrial ultrastructure, and defective quality control. A key question arising from these findings was: which molecular partner(s) mediated the functional effects of MCU?

### MECOM is identified as a functional partner of MCU and establishes a cross-organelle signaling axis in HGG

**Figure 5:**
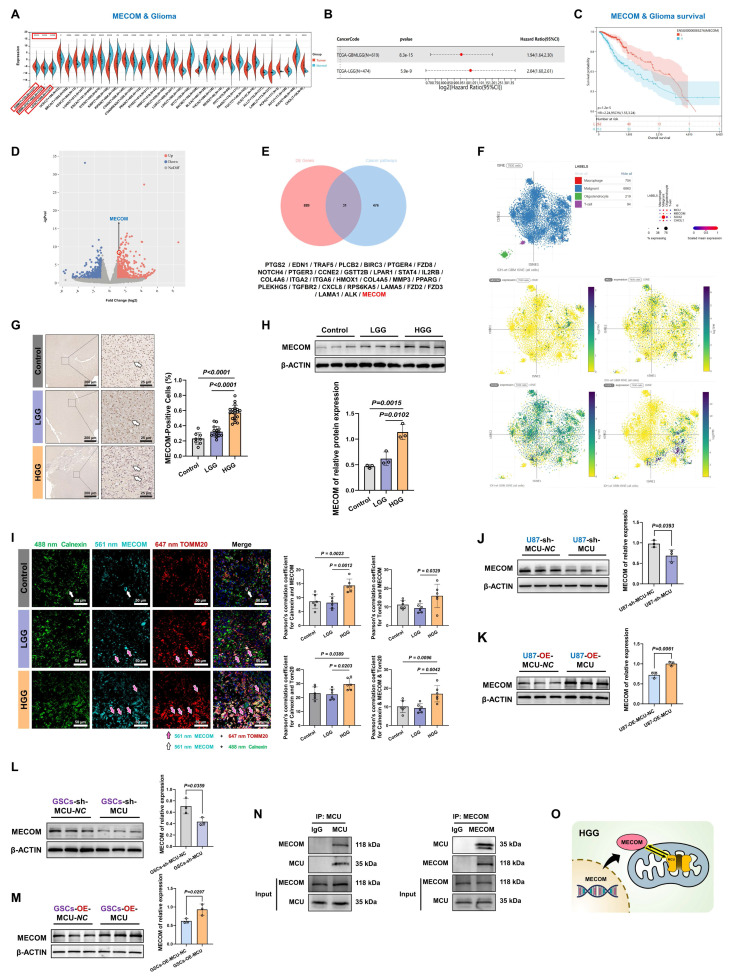
** MECOM is identified as a functional partner of MCU with prognostic significance in glioma. (A)** Pan-cancer bioinformatic analysis of MECOM mRNA expression in tumor (red) versus normal (blue) tissues. The red boxes highlight the glioma cohorts. **(B** and** C)** Prognostic significance of MECOM in glioma, showing the hazard ratio forest plot **(B)** and Kaplan-Meier overall survival curve** (C)** based on TCGA datasets. **(D)** Volcano plot of RNA-seq data illustrating differentially expressed genes in U87MG cells following MCU knockdown, with the downregulation of MECOM specifically highlighted. **(E)** Venn diagram displaying 31 overlapping genes between the MCU knockdown differentially expressed genes and cancer pathway-associated gene sets, identifying MECOM as a key candidate. **(F)** Single-cell RNA-seq (t-SNE) analysis mapping the expression distribution of MCU, MECOM, and glioma stemness markers (SOX2, CD133) across diverse cell populations within the glioblastoma microenvironment. **(G)** IHC images and corresponding quantitative analysis of MECOM expression in Control, LGG, and HGG tissues. **(H)** Western blot analysis and relative protein quantification of MECOM in clinical tissue lysates. **(I)** Representative multi-color confocal immunofluorescence images and quantitative colocalization analysis (Pearson's correlation coefficient) among Calnexin (green), MECOM (cyan), and TOMM20 (red) across clinical grades. **(J** and** K)** Western blot analysis and relative protein quantification of MECOM expression in U87MG cells following MCU knockdown **(J)** and MCU overexpression **(K)**. **(L** and** M)** Western blot analysis and relative protein quantification of MECOM expression in GSCs following MCU knockdown **(L)** and MCU overexpression** (M)**. **(N)** Co-IP assays validating the endogenous physical interaction between MCU and MECOM in U87MG cells. **(O)** Schematic representation illustrating MECOM as a functional interacting partner of MCU at the mitochondria. Data are presented as mean ± SD. *P < 0.05, **P < 0.01, ***P < 0.001, ****P < 0.0001.

To identify key molecular partners within the MCU regulatory network and elucidate the cross-organelle signaling axis in HGG, we comprehensively evaluated MECOM. Pan-cancer bioinformatic screening confirmed the tumor-specific overexpression of MECOM across multiple malignancies, with the most pronounced elevation observed in glioblastoma (Fig. 5A and S7A). This significant upregulation in tumor versus non-tumor tissues was independently confirmed using the GEO dataset (GSE4290) (Fig. S7B). Subsequent TCGA and TARGET survival analyses demonstrated a significantly higher hazard ratio and shortened overall survival for high-MECOM patients in combined GBM-LGG cohorts, which was further validated by Kaplan-Meier survival curves (Fig. 5B and 5C).

To elucidate the upstream-downstream relationship between MCU and MECOM, we performed RNA sequencing two weeks after lentiviral sh-MCU treatment in U87MG cells. This revealed extensive transcriptional reprogramming (Fig. 5D). Functional enrichment analysis demonstrated that these differentially expressed genes were highly enriched in migration- and metastasis-associated programs, including extracellular matrix (ECM)-receptor interactions, focal adhesion, and the PI3K-Akt signaling pathway. Additionally, cellular component analysis highlighted structural reorganizations associated with migration, such as the basement membrane and filopodia (Fig. S7C-E), confirming a direct transcriptomic link between MCU signaling and the tumor migration program. Furthermore, targeted analysis of voltage-gated ion channels (VGICs) revealed that MCU silencing induced complex expression reprogramming: sodium channels (SCN2A, SCN1B) and TRP channels (TRPV2) were significantly upregulated, whereas the calcium channel CACNA2D3 was downregulated, alongside bidirectional modulation of potassium channels (KCNJ15 and KCNN3) (Fig. S7F). This provided additional mechanistic insight into how the MCU axis modulated malignant membrane excitability. Next, we sought to identify the central transcriptional mediators driving this extensive molecular reprogramming. Venn diagram analysis identified 31 overlapping differentially expressed genes associated with cancer pathways, and predictive PPI network analysis highlighted MECOM as a primary candidate within this regulatory network (Fig. 5E and S7G). Importantly, to clarify the cellular origin and subtype specificity of this signaling axis, we mapped MECOM and MCU expression onto human GBM single-cell RNA sequencing datasets (t-SNE/UMAP). This analysis distinctly revealed that the MCU-MECOM axis was predominantly enriched within the malignant cell populations rather than the tumor microenvironment stroma (Fig. 5F).

Clinical validation using human glioma specimens further corroborated these findings. Immunohistochemistry demonstrated more than 2-fold elevated MECOM protein levels in HGG compared to controls, with western blot confirming a malignancy grade-dependent accumulation (Fig. 5G and 5H). Furthermore, immunofluorescence analysis revealed a distinct spatial shift: while MECOM predominantly localized to the endoplasmic reticulum in normal controls, it exhibited prominent mitochondrial outer membrane co-localization with TOMM20 in both LGG and HGG tissues (Fig. 5I).

Functional assays established a robust MCU-MECOM regulatory cascade. Genetic ablation of MCU (sh-MCU) significantly reduced MECOM protein expression in both U87MG and GSCs (Fig. 5J and 5L). Conversely, MCU overexpression (OE-MCU) substantially upregulated MECOM levels (Fig. 5K and 5M). Finally, to determine whether this functional regulation involved physical complex formation, we performed reciprocal Co-IP assays. The results confirmed a direct interaction between MCU and MECOM, with significant mutual enrichment in their respective immunoprecipitates (Fig. 5N and S7H).

This integrated multi-omics profiling identified MECOM as a key transcriptional amplifier within the MCU signaling network, establishing a cross-organelle signaling paradigm that contributes to glioma molecular heterogeneity (Fig. 5O).

### MECOM coordinates MAMs-mediated metabolic reprogramming in HGG

**Figure 6:**
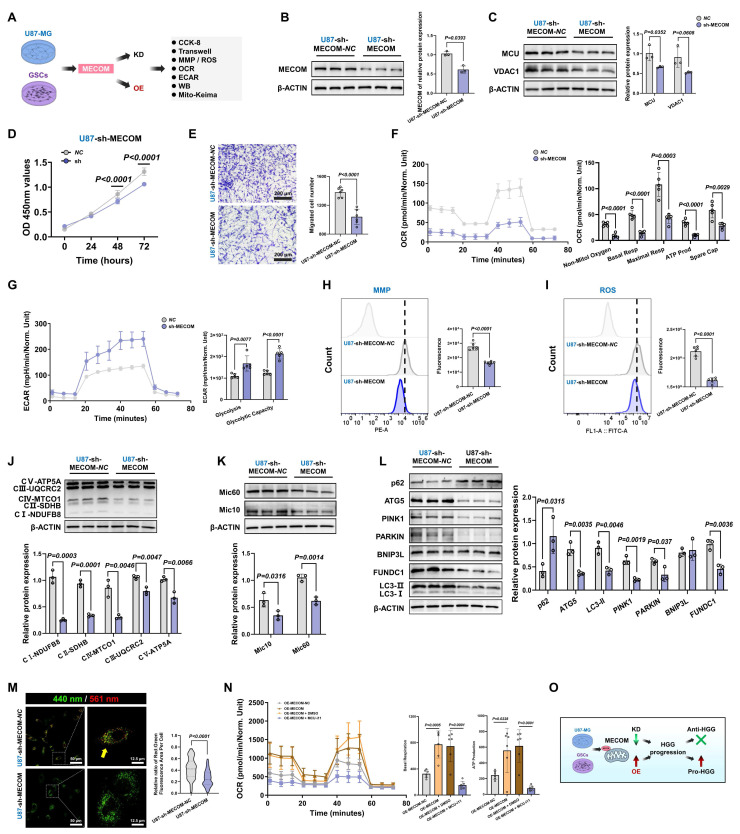
** MECOM coordinates MAMs-mediated metabolic reprogramming and mitochondrial function. (A)** Schematic illustration of the experimental design for lentiviral-mediated MECOM knockdown (KD) and overexpression (OE) in U87MG cells and GSCs, followed by functional assays. **(B)** Western blot analysis and relative protein quantification of MECOM expression in U87MG cells following MECOM knockdown. **(C)** Western blot analysis and relative protein quantification of MCU and VDAC1 in U87MG cells following MECOM knockdown.** (D)** CCK-8 assay assessing the proliferation of U87MG cells following MECOM knockdown. **(E)** Representative images and quantitative analysis of Transwell migration assays for U87MG cells following MECOM knockdown. **(F)** OCR analysis evaluating mitochondrial respiration parameters (non-mitochondrial oxygen, basal respiration, maximal respiration, ATP production, and spare capacity) in U87MG cells following MECOM knockdown.** (G)** ECAR analysis assessing glycolytic function parameters (glycolysis and glycolytic capacity) in U87MG cells following MECOM knockdown. **(H** and** I)** Flow cytometry analysis and corresponding quantification of MMP **(H)** and intracellular ROS levels **(I)** in U87MG cells following MECOM knockdown. **(J)** Western blot analysis and relative protein quantification of OXPHOS complex subunits (CI-CV) in U87MG cells following MECOM knockdown.** (K)** Western blot analysis and relative protein quantification of the MICOS complex subunits Mic10 and Mic60 following MECOM knockdown. **(L)** Western blot analysis and relative protein quantification of key autophagy and mitophagy-related proteins (p62, ATG5, LC3-I/II, PINK1, PARKIN, BNIP3L, and FUNDC1) in U87MG cells following MECOM knockdown. **(M)** Representative confocal images and quantitative analysis of mitophagy flux using the Mito-Keima probe in U87MG cells. **(N)** OCR analysis evaluating mitochondrial respiration in MECOM-overexpressing (OE-MECOM) U87MG cells treated with or without the specific MCU inhibitor, MCU-i11. This demonstrates that pharmacological blockade of MCU abolishes the hyper-metabolic state induced by MECOM overexpression. **(O)** Schematic summary illustrating that MECOM drives HGG progression, highlighting the therapeutic vulnerability of this metabolic circuit. Data are presented as mean ± SD.

Based on the established MCU-MECOM regulatory axis, we developed a graded MECOM expression model in GBM cells to systematically evaluate its functional impact (Fig. 6A). Lentivirus-mediated MECOM knockdown (sh-MECOM) significantly reduced MECOM protein levels in U87MG cells (Fig. 6B). Interestingly, MECOM silencing concurrently reduced MCU expression (Fig. 6C). Together with our earlier finding that MECOM is transcriptionally driven by MCU signaling, this uncovered a robust positive feedback loop: MCU-mediated calcium flux-initiated MECOM upregulation, and MECOM, acting as a transcriptional amplifier, further sustained MCU expression, thereby locking the GBM cells in a hyper-metabolic state.

Functionally, sh-MECOM significantly suppressed malignant phenotypes, decreasing cell viability in CCK-8 assays and reducing transwell migration capacity (Fig. 6D and 6E). Metabolic profiling revealed comprehensive OXPHOS inhibition, with significant decreases in basal respiration, maximal respiratory capacity, and ATP production (Fig. 6F). Compensatory glycolytic capacity was concurrently impaired, establishing a dual metabolic blockade (Fig. 6G). This bioenergetic collapse was accompanied by severe mitochondrial dysfunction, as evidenced by MMP collapse and decreased ROS production (Fig. 6H and 6I).

At the molecular level, Western blot confirmed the concurrent downregulation of OXPHOS complexes (CI-CV), which correlated with diminished expression of the MRC remodeling regulators Mic10 and Mic60 (Fig. 6J and 6K). Furthermore, autophagic flux analysis showed that sh-MECOM significantly reduced ATG5 and LC3-II alongside p62 accumulation, while concurrently suppressing major mitophagy pathways, including PINK1/PARKIN and FUNDC1 (Fig. 6L). Mito-Keima assays validated these results, demonstrating a profound reduction in mitophagy activity (Fig. 6M). Additional evaluations confirmed that sh-MECOM significantly suppressed the MAMs tethering complexes GRP75 and IP3R1, while impairing antioxidant markers SOD1 and GPX4 (Fig. S8A and S8B).

Crucially, to functionally validate whether MECOM drove this metabolic reprogramming *through* MCU, we treated MECOM-overexpressing cells with the specific MCU inhibitor (MCU-i11). Pharmacological blockade of MCU completely abrogated the MECOM-induced enhancement of basal respiration and ATP production (Fig. 6N). This rescue experiment distinctly confirmed that MCU was an essential downstream effector required for MECOM-mediated bioenergetics. Furthermore, MECOM overexpression (OE-MECOM) enhanced malignant phenotypes and mitochondrial function, while consistent molecular reprogramming was observed in GSCs models, collectively substantiating MECOM's vital role in tumor stemness maintenance (Fig. S8C-H and Fig. S9).

Collectively, these data demonstrated that MECOM globally orchestrated metabolic-autophagic networks via an interdependent feedback circuit with MCU (Fig. 6O).

### Autophagy actively drives migration, and clinical evidence supports the MCU-coordinated ultrastructural-metabolic axis

**Figure 7:**
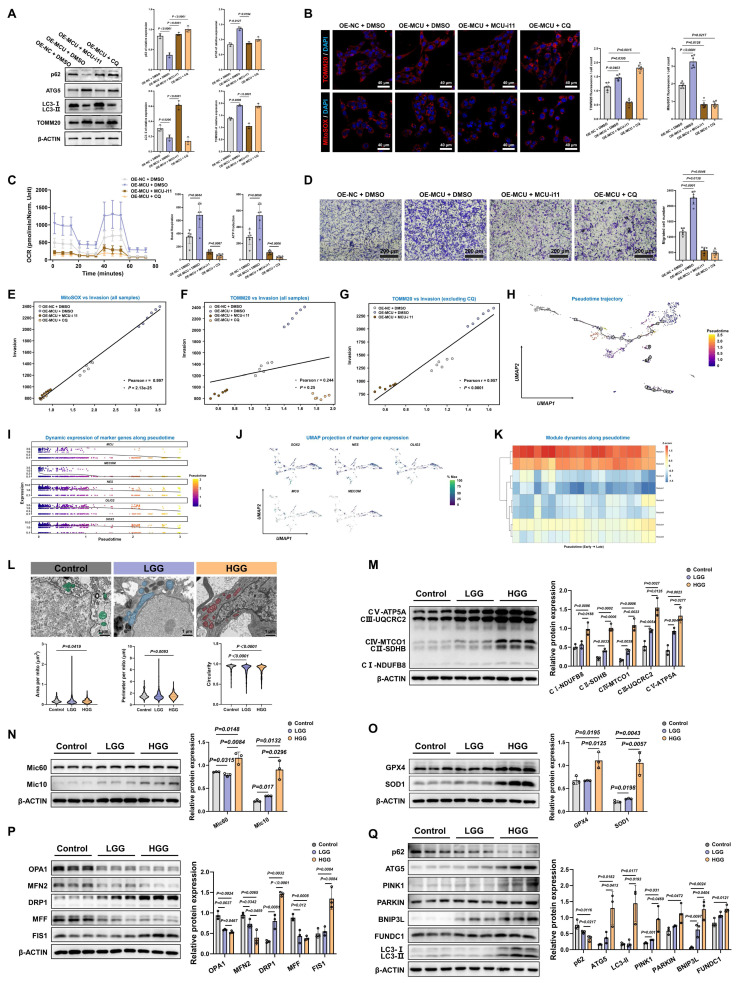
** Mitophagy actively drives MCU-mediated migration, supported by single-cell trajectory and clinical cohort validation. (A)** Western blot analysis and quantification of autophagy markers (p62, ATG5, LC3-I/II) and TOMM20 in MCU-overexpressing (OE-MCU) cells subjected to MCU-i11 or CQ. **(B)** Representative confocal images and quantification of mitochondrial mass (TOMM20, top) and mitochondria-specific ROS (MitoSOX, bottom) under the indicated treatments. Nuclei were counterstained with DAPI. **(C)** OCR analysis demonstrating the rescue of MCU-induced hyper-metabolism by MCU-i11 and the autophagy inhibitor CQ. **(D)** Representative images and quantitative analysis of Transwell migration assays demonstrating that CQ abrogated MCU-driven cell migration. **(E-G)** Pearson correlation analysis evaluating the linear relationship between cell migration capacity and MitoSOX **(E)**, or TOMM20 across all samples **(F)** and excluding the CQ-treated group **(G)**. **(H-K)** Single-cell RNA sequencing (scRNA-seq) pseudotime trajectory analysis. **(H)** UMAP plot illustrating the evolutionary pseudotime trajectory of glioma cells. **(I)** Dynamic expression trends of MCU, MECOM, and classical stemness/lineage markers along the pseudotime.** (J)** UMAP projections showing the expression distribution of these marker genes. **(K)** Heatmap depicting the expression dynamics of co-expressed gene modules from early to late pseudotime stages. **(L)** Ultrastructural evaluation of mitochondria across clinical glioma grades (Control, LGG, HGG). Left: Representative TEM images with false-colored mitochondria (green, blue, red). Right: Quantitative morphological analysis of mitochondrial area, perimeter, and circularity. **(M)** Western blot analysis and relative protein quantification of OXPHOS complex subunits (CI-CV) in Control, LGG, and HGG clinical tissue lysates. **(N)** Western blot analysis and quantification of the MICOS complex subunits Mic60 and Mic10 in clinical tissues. **(O)** Western blot analysis and quantification of antioxidant proteins GPX4 and SOD1 in clinical tissues. **(P)** Western blot analysis and quantification of mitochondrial dynamics-related proteins (OPA1, MFN2, DRP1, MFF, FIS1) in clinical tissues. **(Q)** Western blot analysis and quantification of mitophagy-related proteins (p62, ATG5, PINK1, PARKIN, BNIP3L, FUNDC1, LC3-I/II) in clinical tissues. Data are presented as mean ± SD.

To definitively prove that MCU-driven mitophagy and metabolic reprogramming actively dictated malignant behavior rather than merely accompanying it, we introduced the autophagy inhibitor CQ and MCU-i11 into the MCU-overexpression models. Western blot analysis confirmed that CQ effectively blocked autophagic flux, as evidenced by LC3-II and p62 accumulation, while MCU-i11 suppressed MCU-induced autophagy initiation (Fig. 7A). Consequently, the disruption of autophagy or MCU channel activity abrogated the accelerated clearance of the mitochondrial mass marker TOMM20 and profoundly suppressed mitochondria-specific ROS bursts (Fig. 7B). Functionally, this pharmacological dual-blockade completely dismantled the hyper-OXPHOS state (Fig. 7C) and abolished the enhanced migration ability induced by MCU overexpression (Fig. 7D). Correlation analyses further highlighted a striking positive association between MitoSOX levels and cell migration, underscoring mitochondrial ROS as a critical driver of aggressiveness (Fig. 7E-G).

To map this mechanistic axis onto the developmental continuum of GBM and explore transcriptomic changes across different stages of tumorigenesis, we performed single-cell RNA sequencing pseudotime analysis. The inferred UMAP trajectory revealed a distinct progression path towards highly malignant states (Fig. 7H). Notably, the dynamic expression of *MCU* and *MECOM* closely paralleled those of critical stemness and migration markers, such as *SOX2*, *NES*, and *OLIG2*, along the pseudotime trajectory (Fig. 7I and 7J). Module dynamics further confirmed the coordinated upregulation of these gene sets during the late stages of tumorigenesis, transcriptomically validating the MCU-MECOM axis as a core driver of the invasive/stem-like state transition (Fig. 7K).

To validate the clinical universality of this "mitochondrial fragmentation-mitophagy activation-metabolic burst" axis, we conducted systematic ultrastructural and molecular profiling of clinical glioma specimens. TEM analysis revealed significantly enhanced mitochondrial fission in HGG, characterized by decreased cross-sectional area and perimeter, and increased circularity (Fig. 7L). Molecular profiling corroborated this hyper-metabolic state. HGG tissues exhibited a universal overexpression of OXPHOS complexes and cristae remodeling regulators (Fig. 7M and 7N). This was accompanied by adaptive antioxidant defense activation, evidenced by significantly elevated GPX4 and SOD1, neutralizing OXPHOS-derived ROS to sustain malignant proliferation (Fig. 7O). Furthermore, the mitochondrial dynamic balance shifted toward severe hyper-fission (upregulated DRP1 and FIS1; downregulated OPA1, MFN2, and MFF), while hyperactivated mitophagy pathways (ATG5, LC3-II, PINK1, PARKIN, BNIP3L, and FUNDC1) were prominently upregulated to maintain mitochondrial quality control (Fig. 7P and 7Q).

To further substantiate the prognostic and translational relevance of this ultrastructural-metabolic axis, we evaluated a core MAMs and mitophagy gene signature (incorporating *HSPA9/GRP75*, *ATG5*, *BNIP3L*, *FUNDC1*, and *VDAC1*) using the extended TCGA and CGGA cohorts. Consistent with our *in vitro* findings, these components were universally upregulated in both GBM and LGG compared to normal tissues. Survival analyses indicated that high expression of *ATG5*, *BNIP3L*, *FUNDC1*, and *VDAC1* strongly correlated with poorer overall survival, whereas *HSPA9* high expression was associated with better survival outcomes (Fig. S10A-O). Moreover, by integrating these key signatures via machine learning-based LASSO-Cox regression, we constructed a robust risk model in the CGGA cohort. This multiparametric signature yielded high predictive accuracy (1-year AUC = 0.588, 3-year AUC = 0.665, 5-year AUC = 0.698) and served as an independent prognostic factor across glioma grades after adjusting for classical clinical covariates (Fig. S10P-S.) These large-scale transcriptomic validations firmly positioned the MCU-MECOM/MAMs network as a clinically actionable diagnostic and prognostic axis.

Therefore, MCU overexpression enhanced MAMs expanding, inducing calcium retrograde transport and mitochondrial calcium overload in HGG. This significantly activated mitochondrial fission and mitophagy, while high expression of Mic10/Mic60 mediated MRC remodeling, thereby elevating electron transfer efficiency and driving tumor metabolic reprogramming. MECOM, as the core transcriptional amplifier of MCU, cooperatively regulated this process. Based on this mechanism, synchronous targeting of MCU elicited a therapeutic “bioenergetic crisis”: metabolic suppression through OXPHOS/glycolysis blockade, structural disintegration via disrupted mitochondrial dynamics, and inhibited mitophagy-mediated inner membrane remodeling, ultimately inducing tumor cell death.

### AI-driven quantification of MAMs ultrastructure enables accurate glioma stratification

To leverage the characteristic changes in the subcellular localization and spatial configuration of MAMs in high-grade gliomas, we developed a dual-stage artificial intelligence framework, MAMs-Net. This framework was designed to identify MAMs structures and quantify their morphological parameters to predict glioma pathological grading from TEM images, strictly following the diagnostic rationale of subcellular structure localization preceding morphological quantification.

**Figure 8:**
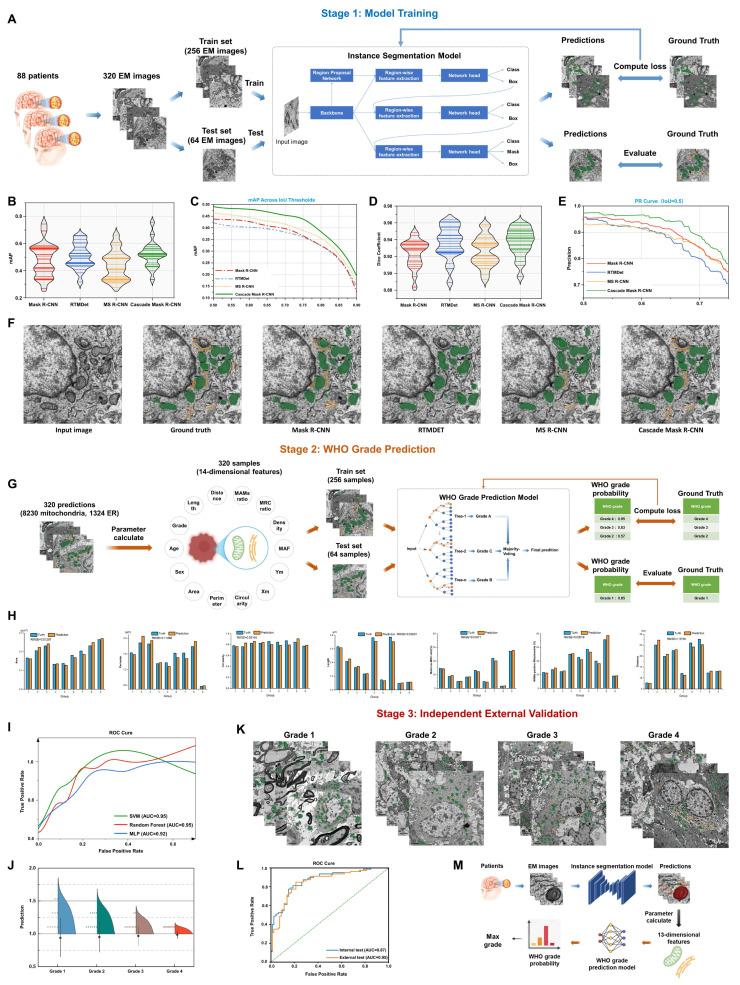
** MAMs-Net, an AI-driven ultrastructural quantification system, enabled accurate glioma stratification. (A)** Schematic workflow of Stage 1: Instance segmentation model training and testing using 320 TEM images from clinical cohorts. **(B-E)** Comparative performance evaluation of four instance segmentation architectures (Mask R-CNN, RTMDet, MS R-CNN, and Cascade Mask R-CNN) based on mean mAP distributions **(B)**, mAP across Intersection over Union (IoU) thresholds **(C)**, Dice coefficients** (D)**, and Precision-Recall (PR) curves at IoU=0.5 **(E)**. **(F)** Representative visual comparison of automated organelle segmentation (mitochondria in green, MAMs/ER in orange) by the four AI models versus manual ground truth annotation. **(G)** Schematic workflow of Stage 2: WHO grade prediction. The framework extracts a 14-dimensional feature vector encompassing ultrastructural morphometrics and MAMs spatial interactions to train classification algorithms. **(H)** Quantitative evaluation showing the consistency between manual annotations (Ground Truth) and AI predictions across key ultrastructural parameters (e.g., area, perimeter, circularity, and MAMs contact metrics). **(I)** ROC curves evaluating the predictive performance of different machine learning classifiers (SVM, Random Forest, and MLP) in the internal test set. **(J)** Distribution (raincloud plots) of the AI-predicted probabilities across actual WHO Grades (1 to 4). **(K)** Representative TEM images from Stage 3: Independent external validation cohort, with automated segmentation overlaid across WHO Grades 1-4. **(L)** ROC curves comparing the diagnostic performance (AUC) of the optimized MAMs-Net in the internal test set versus the independent external validation set. **(M)** Summarized clinical translation pipeline of the MAMs-Net diagnostic framework.

The first stage employed instance segmentation technology to precisely identify individual mitochondria and endoplasmic reticulum instances. The initial organelle recognition and morphometric measurement dataset comprised 320 TEM images from 88 patients, randomly divided into training and independent test sets at a 4:1 ratio. Data augmentation strategies, including random scaling and flipping, were implemented during training to enhance model generalization (Fig. 8A). During model evaluation, Cascade Mask R-CNN demonstrated superior performance compared to Mask R-CNN, RTMDet, and MS R-CNN. It consistently achieved the highest mean average precision (mAP) across varying intersection over union (IoU) thresholds (Fig. 8B and 8C). Furthermore, it exhibited excellent boundary alignment, as indicated by the Dice coefficient, and significantly reduced false positive rates, as measured by precision metrics (Fig. 8D and 8E). Visual assessments of the segmentation outputs confirmed its superior accuracy in identifying organelle instances (Fig. 8F). Consequently, Cascade Mask R-CNN was chosen as the optimal solution for high-precision organelle localization.

The second stage extracted pathologically significant quantitative ultrastructural features from segmentation outputs. Machine learning modeling established mappings between these feature patterns and WHO glioma grading standards. Quantitative morphological features were extracted from segmentation results during the grading prediction phase. Fourteen discriminative parameters were selected by integrating patient sex, patient age, mitochondrial morphological parameters from TEM images, and spatial interaction characteristics of MAMs: (1) WHO grade, (2) age, (3) gender, (4) individual mitochondrial area, (5) individual mitochondrial perimeter, (6) individual mitochondrial circularity, (7) mitochondrial centroid X-coordinate, (8) mitochondrial centroid Y-coordinate, (9) MAF, (10) single-cell mitochondrial density, (11) matrix-to-MRC ratio, (12) MAMs-positive mitochondria, (13) vertical distance between MAMs structures, (14) contact length between MAMs structures. Feature vectors containing mean values, standard deviations, maximum and minimum values were generated per image by aggregating all instance data. The feature dataset was then divided into training and test sets using a 4:1 ratio (Fig. 8G). Comparative analysis against manual measurements identified seven optimal mitochondrial morphology and MAMs spatial interaction features: (1) individual mitochondrial area, (2) individual mitochondrial perimeter, (3) individual mitochondrial circularity, (4) matrix-to-MRC ratio, (5) MAMs-positive mitochondria, (6) vertical distance between MAMs structures, (7) contact length between MAMs structures (Fig. 8H). Comparative analysis between ground truth and predicted values validated the accuracy of key discriminative features, including individual mitochondrial area, perimeter, circularity, matrix-to-MRC ratio, and MAMs contact lengths. For grading prediction, classifier comparison among Random Forest (RF), Support Vector Machine (SVM), and Multilayer Perceptron (MLP) revealed that both RF and SVM achieved an outstanding F1 score (AUC = 0.95) on the test set, outperforming the MLP model (AUC = 0.92) (Fig. 8I). RF was subsequently selected as the core algorithm, demonstrating robust prediction distributions across WHO grades (Fig. 8J).

Crucially, to rigorously assess the clinical generalizability of MAMs-Net, we introduced a Stage 3 independent external validation cohort. The established model was directly applied to predict the grades of external TEM images from new glioma patients (Grades 1 to 4) (Fig. 8K). Strikingly, the model maintained an exceptional diagnostic performance with an external test AUC of 0.95, which was highly consistent with and even surpassed the internal test performance (Fig. 8L). Moreover, cross-species segmentation generalizability was also confirmed using the independent Lucchi++ dataset.

This integrated system established an end-to-end diagnostic pipeline progressing from raw TEM image input through subcellular recognition and quantitative feature extraction to final WHO grade output. It presented a novel computer-aided diagnostic methodology with substantial translational potential for glioma pathological grading (Fig. 8M).

## Discussion

Our study delineated a novel signaling axis in glioblastoma, wherein the MCU-MECOM positive feedback loop drove malignancy through structural and functional reprogramming of mitochondria. We demonstrated that MCU overexpression expanded MAMs interfaces, triggering calcium-dependent mitochondrial ultrastructural remodeling, and identified MECOM as a key transcriptional amplifier within this pathway. Furthermore, we translated these mechanistic insights into a diagnostic tool, MAMs-Net, which automated the quantification of MAMs ultrastructure for glioma stratification.

First, we revealed MCU-driven MAMs-dependent pathological restructuring where MCU overexpression enhanced MAMs anchoring to induce calcium retrograde transport, causing mitochondrial calcium overload and Mic10/Mic60-mediated MRC remodeling. This cycle coordinated metabolic reprogramming to sustain tumor stemness, a dynamic transition distinctly captured by our time-resolved pharmacological profiling. Second, we identified a vicious cross-organelle malignancy circuit: MCU-mediated calcium flux-initiated MECOM upregulation, while MECOM, acting as a transcriptional amplifier, sustained MCU expression. Third, we proposed a novel “bioenergetic crisis” therapeutic strategy wherein targeting MCU synchronously triggered synergistic structural disintegration, autophagic arrest and metabolic suppression. Finally, leveraging these discoveries, we developed and externally validated an AI-powered MAMs ultrastructural quantification system (MAMs-Net).

While previous studies have individually implicated MCU in targeted nanosystem therapies for GBM^14^ and MECOM in promoting glioma cell proliferation^15^, the precise spatiotemporal dynamics and the structural-metabolic coupling mechanisms linking these factors remained elusive. Our study significantly advances the field by delineating a novel "calcium signaling-structural remodeling-transcriptional reprogramming" paradigm. Specifically, our work provides three unprecedented mechanistic insights. First, we discovered a robust positive feedback loop between MCU and MECOM; MCU-mediated calcium flux initiates MECOM upregulation, while MECOM acts as a transcriptional amplifier to sustain MCU expression, thereby locking the cells in a hyper-metabolic state. Second, we elucidated the MAMs-cristae ultrastructural axis. We demonstrated that MCU-driven calcium overload in GBM does not induce apoptosis, but rather triggers adaptive mitochondrial cristae remodeling and quality control to buffer ROS and sustain high OXPHOS. Third, through time-resolved functional assays and autophagy-rescue experiments (using si-ATG5 and chloroquine), we provided direct causal evidence that mitophagy actively drives tumor migration, resolving the "pro-survival paradox" of calcium overload in GBM.

The mitochondrial contact site and cristae-organizing system (MICOS) localizes at cristae junctions to orchestrate cristae formation. MICOS comprises Mic60 subcomplexes (Mic60/Mic19/Mic25 subunits maintaining cristae junction integrity and inter-membrane stability) and Mic10 subcomplexes (Mic10/Mic26/Mic27 subunits regulating cristae morphology and stability)^32^-^35^. Pei-I Tsai's team demonstrated that PINK1-regulated Mic60 overexpression drove dynamic cristae remodeling across subcellular compartments to restore ATP production and cellular homeostasis^36^. In colorectal adenocarcinoma, GBM, breast cancer, prostate cancer, and lung adenocarcinoma, Mic60 downregulation significantly suppresses cellular proliferation; further investigations revealed that Mic60-mediated repair of acutely damaged “ghost mitochondria” exhibited tumor-promoting pathological potential^37^. In this study, MCU overexpression in HGG induced mitochondrial calcium overload triggering coordinated mitophagy-fission cascades: calcium-dependent PARKIN translocation initiated p62-mediated mitophagy concurrent with calcium-activated DRP1-driven mitochondrial fragmentation. Fragmented mitochondria underwent Mic10/Mic60-centric MICOS-mediated cristae restructuring, significantly enhancing respiratory efficiency and providing key evidence for cristae-respiration coupling. Specifically, Mic10/Mic60-dependent restructuring optimized respiratory chain spatial topology and supercomplex assembly efficiency in HGG. This discovery complemented the PINK1-Mic10/Mic60 axis theory of mitochondrial dynamic remodeling while expanding pathological models linking MAMs integrity loss to organ dysfunction^38^.

MAMs represent the most abundant membrane contact sites between organelles in numerous cell types. Beyond serving as specialized subcellular signaling platforms for exchanging metabolites such as calcium and glycerophospholipids, MAMs function as critical hubs for essential physiological processes including mitochondrial fission, mitochondrial DNA replication, and autophagosome biogenesis^9^,^22^. Substantial evidence has implicated intracellular calcium fluctuations, calcium uniporters and calcium-signaling activation in carcinogenesis^13^,^39^. For instance, reducing intracellular calcium levels significantly suppresses migration and invasion in hepatocellular, breast, and lung carcinomas^40^. MCU-mediated calcium influx promotes mitochondrial biogenesis and colon cancer proliferation^41^, while MCU knockout impairs calcium uptake to inhibit embryonal rhabdomyosarcoma growth^42^. This study identified MCU-mediated mitochondrial calcium overload as the initiating event driving malignant progression in gliomas. Recent work demonstrated that modulating ER calcium transients at MAMs triggered autophagosome formation^43^. Notably, calcium antagonists reduce autophagy to enhance temozolomide sensitivity in GBM, suppressing tumor growth and extending survival in tumor-bearing mice^44^. *Nature* has reported autophagy as a peripheral mitochondrial fission process eliminating damaged compartments^45^. In this work, MCU overexpression was found to correlate positively with HGG oncogenesis. Mechanistically, this calcium-coupled mitophagy-fission axis enabled continuous ultrastructural remodeling. Crucially, addressing the long-standing controversy of whether autophagy is a mere bystander or an active driver in tumor progression, our rescue experiments using *ATG5* silencing and chloroquine unequivocally demonstrated that disrupting autophagic flux directly abrogated MCU-induced cell migration and mitochondrial ROS bursts. This confirmed a causal relationship where targeted mitophagy actively drives the malignant phenotype. Furthermore, our novel mitochondrial transfer experiments—where mitochondria isolated from MCU-deficient cells failed to support ATP production in normal astrocytes—solidified the concept that MCU dysregulation leaves an intrinsic, transmissible pathological imprint on organelle function. This structural-functional co-evolution amplifies OXPHOS capacity while sustaining glycolytic compensation, ultimately creating a highly aggressive metabolic environment. This mechanism supports organelle pathology-mediated apoptosis resistance in GBM^46^.

Existing studies have reported MECOM promotes tumor progression in ovarian cancer, lung squamous cell carcinoma, and acute myeloid leukemia^47^-^49^. To our knowledge, no data existed on functional interdependency between MCU and MECOM in HGG progression. Here, we provided definitive evidence delineating a unique MCU-MECOM positive feedback axis. We discovered that MCU acted as an essential downstream effector for MECOM-induced proliferation and bioenergetic metabolism; specifically, the pharmacological blockade of MCU using MCU-i11 completely nullified the hyper-metabolic state induced by MECOM overexpression. Conversely, MECOM functioned as a critical transcriptional amplifier that sustained MCU expression under increased mitochondrial calcium uptake. This mutually reinforcing circuit represents a superior therapeutic vulnerability, as it locks glioma cells in a hyper-metabolic state that can be catastrophically dismantled by stimulus-induced cancer cell death strategies.

Beyond driving bulk metabolic shifts, our data supported a model whereby HGG leveraged expanded MAMs architecture to activate a quality control mechanism of selective mitophagy. We posited that calcium overload, facilitated by reinforced ER-mitochondria contacts, not only triggered mitochondrial fragmentation but also promoted the selective elimination of mitochondrial subpopulations with low MICOS complex levels. This process enriches the cellular pool in functionally superior, Mic60/Mic10-high mitochondria, which are primed for efficient cristae remodeling and OXPHOS. This model elucidated the "pro-survival paradox" of MICOS complexes in cancer: by clearing dysfunctional units and amplifying the respiratory capacity of the remaining network, the MCU-MECOM axis establishes a feed-forward loop that sustains the high-energy demands of aggressive tumors.

This study revealed pathological MAMs ultrastructural remodeling as the nexus connecting MCU-mediated calcium signaling to mitochondrial functionality: TEM images have first demonstrated that MCU overexpression directly expanded mitochondria-ER contact interfaces to drive calcium-dependent cristae restructuring, providing structural-functional validation for the “tumor metabolic switch” hypothesis^50^-^52^. Concurrently, the MCU-MECOM cross-compartmental regulatory circuit has overturned conventional paradigms—MCU not only functioned as an ion channel but acted as a transcriptional regulator of MECOM, offering molecular explanations for mitochondrial retrograde signaling^53^,^54^. Addressing glioma's unique fragmentation-autophagy-metabolic burst trinity phenotype, we identified MCU as the master upstream target. Our single-cell pseudotime trajectory analysis corroborated this at the transcriptomic level, elegantly mapping the coordinated hyperactivation of the MCU-MECOM axis alongside classic stemness and migration markers during the dynamic evolution of GBM. Leveraging these biological insights, we innovatively developed the MAMs-Net AI system for automated ultrastructural quantification. Unlike previous preliminary models, MAMs-Net underwent rigorous independent external validation, achieving an exceptional AUC of 0.95 on external clinical cohorts and demonstrating cross-species generalizability. It is a hypothesis-driven tool tightly integrated with a defined biological mechanism, moving beyond mere pattern recognition to provide pathophysiologically interpretable insights. Building upon this cascade, future clinical translation will focus on synergistic therapies targeting both MCU-MAMs signaling and cristae destabilization, integrated with spatial omics to resolve intratumoral heterogeneity and advance organelle interaction-guided precision oncology^55^.

While this study employed comprehensive functional assays—including pharmacological rescue, genetic silencing, time-resolved tracking, and mitochondrial transfer—to firmly establish the MCU-MECOM causal network, some limitations remain. Although our quantitative electron microscope dataset is robust and MAMs-Net achieved high accuracy on an independent external cohort, its broad clinical utility and integration into standard pathological workflows would benefit from large-scale, multi-center prospective trials. Additionally, while we validated the core findings across U87MG and patient-derived GSCs, the extreme inter- and intra-tumoral heterogeneity of GBM suggests that future studies utilizing spatial transcriptomics could further elucidate the exact tumor microenvironmental niches where this MAMs-dependent metabolic rewiring is most pronounced^56^.

This study revealed that MCU overexpression induced mitochondrial calcium overload through enhanced MAMs, triggering Mic10/Mic60-mediated cristae restructuring and metabolic reprogramming in glioma. We discovered an MCU-MECOM regulatory loop where MCU upregulated MECOM transcription, creating a tumor-promoting circuit. Targeting MCU simultaneously disrupted metabolism, structure and autophagy, inducing synergistic anti-tumor effects. We further developed MAMs-Net, an AI system for automated MAMs quantification, advancing glioma diagnosis. These findings established new paradigms for understanding organelle networks in cancer and provided novel therapeutic and diagnostic strategies.

## Supplementary Material

Supplementary figures and tables.

## Data Availability

Further information and requests for resources, reagents, mouse lines, and all original code used in this study should be directed to and will be fulfilled by the lead contact, Yayun Wang (Email: wangyy@fmmu.edu.cn). All data reported in this paper and any additional information required to reanalyze the data are available from the lead contact upon request.

## Abbreviations

2-DG: 2-deoxyglucose
AI: Artificial intelligence
ATG5: Autophagy-related protein 5
ATP: Adenosine triphosphate
AUC: Area under the curve
BCA: Bicinchoninic acid
bFGF: Basic fibroblast growth factor
BNIP3L: BCL2 interacting protein 3 like
BSA: Bovine serum albumin
CCCP: Carbonyl cyanide 3-chlorophenylhydrazone
CCK-8: Cell counting kit-8
CGGA: Chinese Glioma Genome Atlas
CNS: Central nervous system
Co-IP: Co-Immunoprecipitation
CQ: Chloroquine
DAB: 3,3'-Diaminobenzidine
DAPI: 4',6-diamidino-2-phenylindole
DEGs: Differentially expressed genes
DMEM: Dulbecco's Modified Eagle's medium
DPBS: Dulbecco's phosphate-buffered saline
DRP1: Dynamin-1-like protein
ECAR: Extracellular acidification rate
ECL: Enhanced chemiluminescence
EGF: Epidermal growth factor
ER: Endoplasmic reticulum
FBS: Fetal bovine serum
FCCP: Carbonyl cyanide 4-(trifluoromethoxy) phenylhydrazone
FDR: False discovery rate
FIS1: Fission protein 1
FUNDC1: FUN14 domain containing 1
GBM: Glioblastoma
GEO: Gene Expression Omnibus
GO: Gene Ontology
GPX4: Glutathione peroxidase 4
GRP75: Glucose-regulated protein 75
GSCs: Glioma stem cells
GSEA: Gene Set Enrichment Analysis
GTEx: Genotype-Tissue Expression
HGG: High-grade glioma
HRP: Horseradish peroxidase
IF: Immunofluorescence
IHC: Immunohistochemistry
IoU: Intersection over Union
IP3R1: Inositol 1, 4, 5-trisphosphate receptor type 1
KEGG: Kyoto Encyclopedia of Genes and Genomes
LASSO: Least absolute shrinkage and selection operator
LC3-II: Microtubule-associated protein 1 light chain 3-II
LGG: Low-grade glioma
MAF: Mitochondrial area fraction
MAMs: Mitochondria-associated membranes
mAP: Mean average precision
Mask R-CNN: Mask Region-based Convolutional Neural Network
MCU: Mitochondrial calcium uniporter
MECOM: MDS1 and EVI1 complex locus
MEM: Minimum essential medium
MFF: Mitochondrial fission factor
MFN2: Mitochondrial fusion protein 2
MICOS: Mitochondrial contact site and cristae-organizing system
Mic10: MICOS complex subunit MIC10
Mic60: MICOS complex subunit MIC60
MiNA: Mitochondrial Network Analysis
MLP: Multi-layer perceptron
MMP: Mitochondrial membrane potential
MOI: Multiplicity of infection
MRC: Mitochondrial respiratory cristae
MS R-CNN: Mask Scoring R-CNN
NAC: N-acetyl-L-cysteine
NCG: NOD/ShiLtJGpt-Prkdcem26Cd52Il2rgem26Cd22/Gpt
NHA: Normal human astrocytes
OCR: Oxygen consumption rate
OPA1: Optic atrophy 1
OS: Overall survival
OXPHOS: Oxidative phosphorylation
p62: Sequestosome-1
PARKIN: Parkin RBR E3 ubiquitin-protein ligase
PBS: Phosphate-buffered saline
PFA: Paraformaldehyde
PINK1: PTEN induced putative kinase 1
PPI: Protein-protein interaction
RF: Random forest
ROS: Reactive oxygen species
RTMDet: Rotated Target Detection
scRNA-seq: Single-cell RNA sequencing
SD: Standard deviation
siRNA: Small interfering RNA
SMOTE: Synthetic Minority Over-sampling Technique
SOD1: Superoxide dismutase 1
SOX2: SRY-related HMG-box 2
STR: Short tandem repeat
SVM: Support vector machine
TARGET: Therapeutically Applicable Research to Generate Effective Treatments
TBHP: Tert-butyl hydroperoxide
TCA cycle: Tricarboxylic acid cycle
TCGA: The Cancer Genome Atlas
TEM: Transmission electron microscope
t-SNE: T-distributed stochastic neighbor embedding
UCSC: University of California, Santa Cruz
UMAP: Uniform Manifold Approximation and Projection
VDAC1: Voltage-dependent anion channel 1
WHO: World Health Organization
