# Supplementary material for: The MCU-MECOM Axis Orchestrates Glioblastoma Progression by Remodeling Mitochondrial Dynamics and Quality Control via MAMs
Source: Int J Biol Sci. 2026 May 1;22(10):5054–82. doi: 10.7150/ijbs.127940 (PMC13215065; doi:10.7150/ijbs.127940)
Supplement: Supplementary file 1 — Supplementary figures and tables. [file ijbsv22p5054s1.pdf]

## Supplementary figures

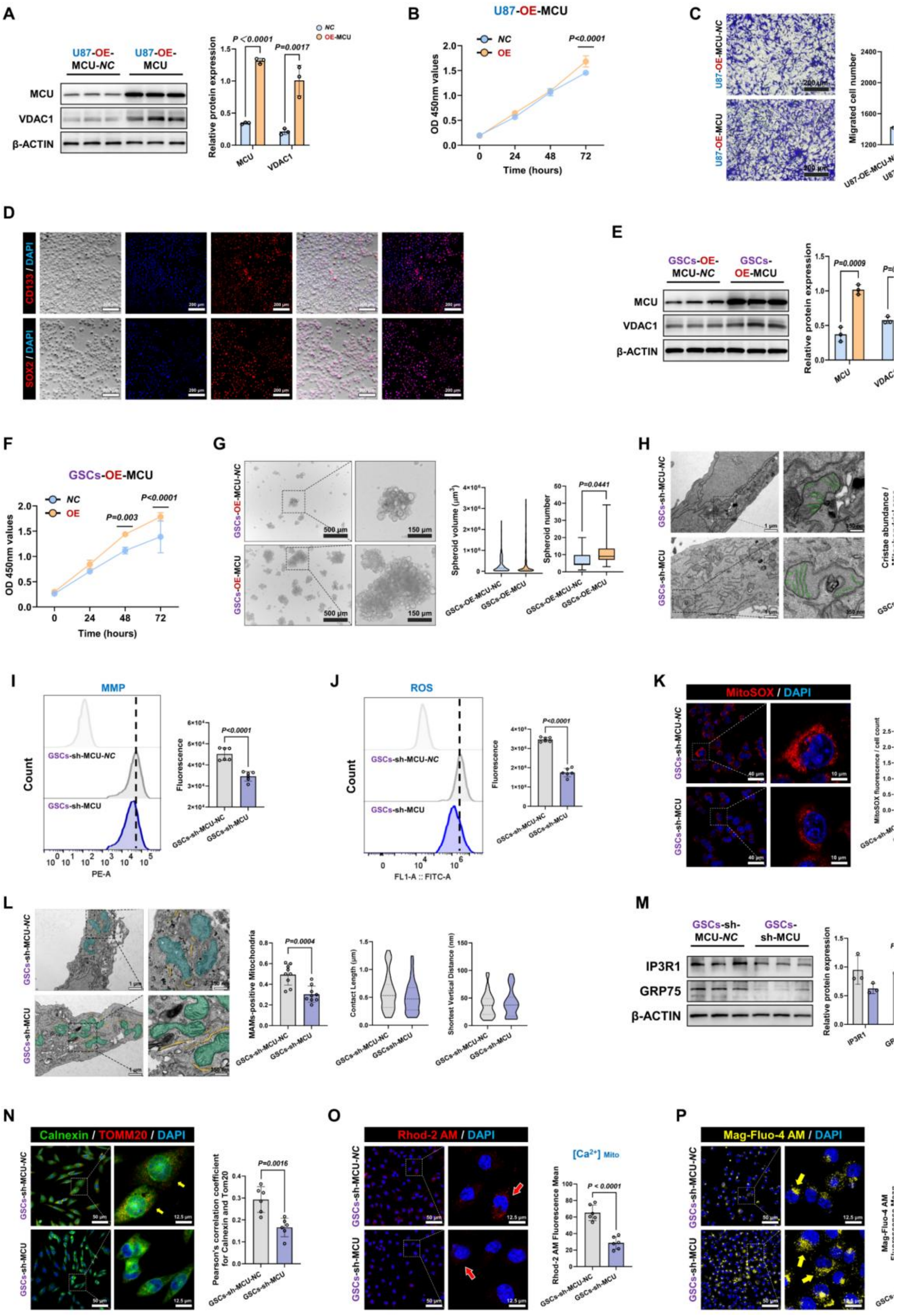


**Figure S1. Characterization of GSCs and in vitro validation of MCU**

**manipulation effects. (A)** Western blot analysis and relative protein quantification of MCU and VDAC1 in U87MG cells following MCU overexpression (OE-MCU). **(B)** CCK-8 proliferation assay of U87MG cells following MCU overexpression. **(C)** Representative images and quantitative analysis of Transwell migration assays for U87MG cells following MCU overexpression. **(D)** Representative immunofluorescence images validating the expression of GSCs markers CD133 (red, top) and SOX2 (red, bottom) in cultured GSCs. Nuclei were counterstained with DAPI (blue). **(E)** Western blot analysis and relative protein quantification of MCU and VDAC1 in GSCs following MCU overexpression. **(F)** CCK-8 proliferation assay of GSCs following MCU overexpression. **(G)** Representative images and statistical analysis of tumorsphere formation by GSCs following MCU overexpression. **(H)** Representative TEM images and quantitative analysis of MRC abundance (highlighted in green) in GSCs following MCU knockdown (sh-MCU). Analyzed mitochondria numbers: sh-MCU-NC (n = 105), sh-MCU (n = 110). **(I** and **J)** Flow cytometry analysis and corresponding quantification of MMP **(I)** and intracellular ROS levels **(J)** in GSCs following MCU knockdown. **(K)** Representative confocal images and quantitative analysis of mitochondria-specific superoxide production using MitoSOX (red) in GSCs following MCU knockdown. **(L)** Ultrastructural analysis of MAMs in GSCs via TEM. Left: Representative images showing mitochondria (green/blue) and ER (yellow) contacts. Right: Quantitative analysis of MAMs-positive mitochondria, contact length, and shortest vertical distance. Analyzed mitochondria numbers: sh-MCU-NC (n = 51), sh-MCU (n = 32). **(M)** Western blot

analysis and relative protein quantification of MAMs tethering proteins IP3R1 and GRP75 in GSCs following MCU knockdown. **(N)** Representative confocal immunofluorescence images and quantitative colocalization analysis of Calnexin (green) and TOMM20 (red) in GSCs following MCU knockdown. **(O** and **P)** Representative confocal images and quantification of mitochondrial calcium levels using Rhod-2 AM (red, **O**) and ER calcium levels using Mag-Fluo-4 AM (yellow, **P**) in GSCs following MCU knockdown. Data are presented as mean ± SD.

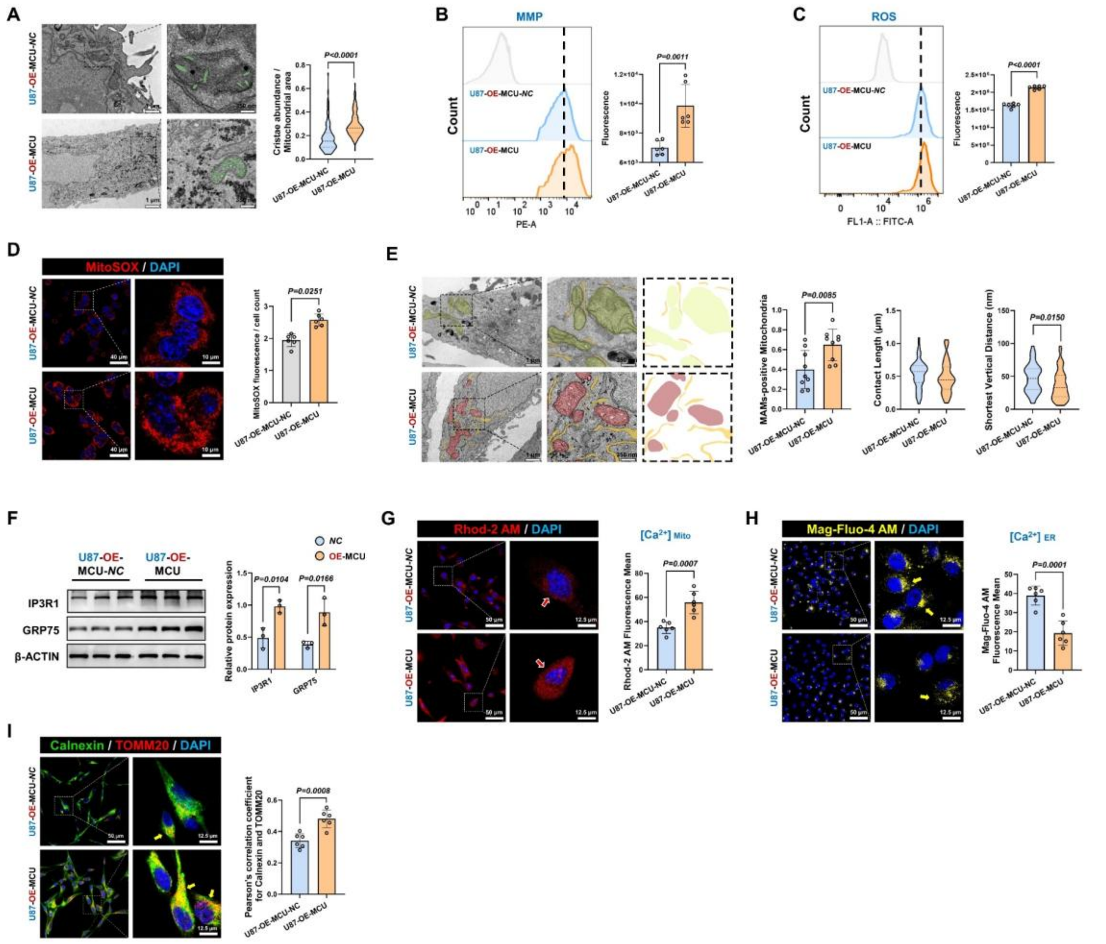

**Figure S2. MCU overexpression drives mitochondrial ultrastructural remodeling and MAMs reinforcement in U87MG. (A)** Representative TEM images and quantitative analysis of MRC abundance (highlighted in green) in U87MG cells following MCU overexpression (OE-MCU). **(B** and **C)** Flow cytometry analysis and corresponding quantification of MMP **(B)** and intracellular ROS levels **(C)** in U87MG cells following MCU overexpression. **(D)** Representative confocal images and quantitative analysis of mitochondria-specific superoxide production using MitoSOX (red) in U87MG cells following MCU overexpression. **(E)** Ultrastructural analysis of MAMs in U87MG cells via TEM. Left: Representative images showing mitochondria and ER contacts. Right: Quantitative analysis of MAMs-positive mitochondria,

contact length, and shortest vertical distance. **(F)** Western blot analysis and relative protein quantification of MAMs tethering proteins IP3R1 and GRP75 in U87MG cells following MCU overexpression. **(G** and **H)** Representative confocal images and quantification of mitochondrial calcium levels using Rhod-2 AM (red, **G**) and ER calcium levels using Mag-Fluo-4 AM (yellow, **H**) in U87MG cells following MCU overexpression. **(I)** Representative confocal immunofluorescence images and quantitative colocalization analysis of Calnexin (green) and TOMM20 (red) in U87MG cells following MCU overexpression. Data are presented as mean ± SD.

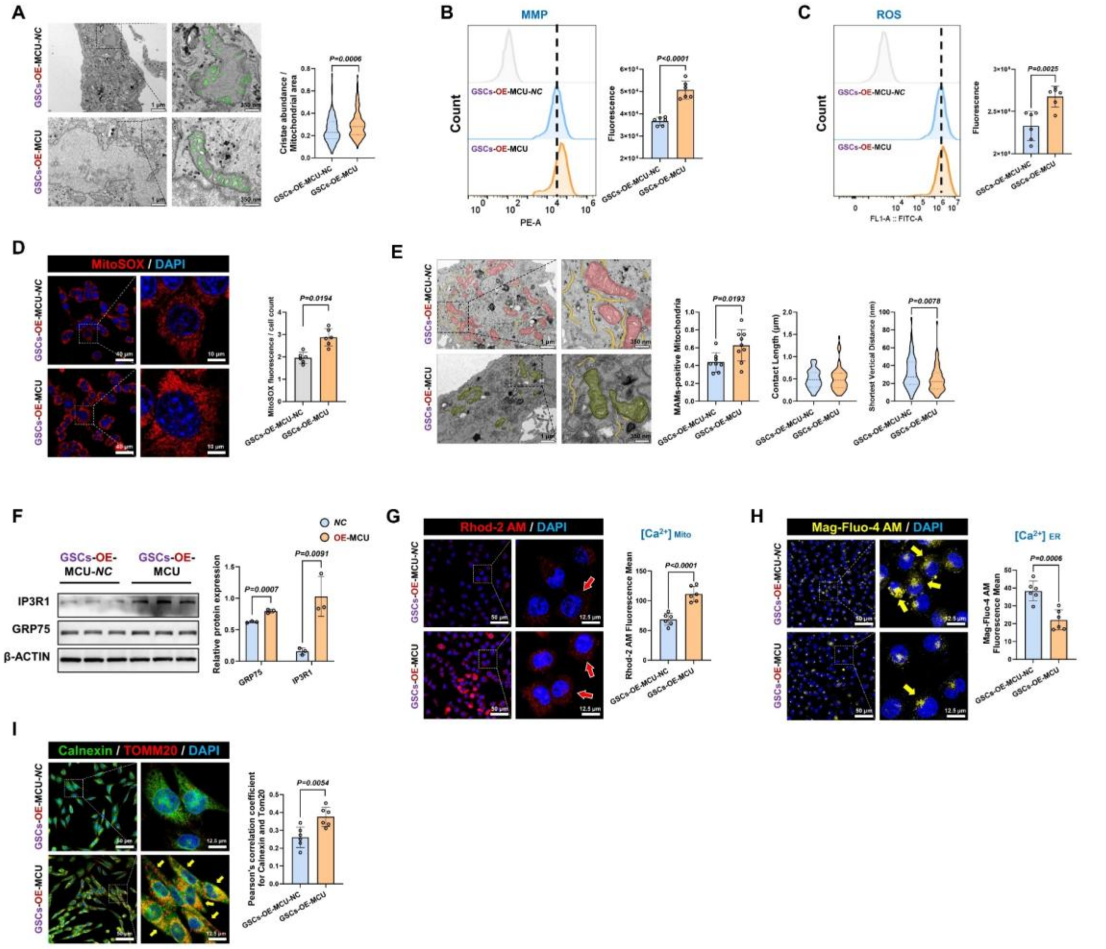


**Figure S3. MCU overexpression drives mitochondrial ultrastructural remodeling and MAMs reinforcement in GSCs. (A)** Representative TEM images and quantitative analysis of MRC abundance (highlighted in green) in GSCs following OE-MCU. **(B and C)** Flow cytometry analysis and corresponding quantification of MMP **(B)** and intracellular ROS levels **(C)** in GSCs following OE-MCU. **(D)** Representative confocal images and quantitative analysis of mitochondria-specific superoxide production using MitoSOX (red) in GSCs following OE-MCU. **(E)** Ultrastructural analysis of MAMs in GSCs via TEM. Left: Representative images showing mitochondria and ER contacts. Right: Quantitative analysis of MAMs-positive mitochondria, contact length, and shortest vertical distance. **(F)** Western blot

analysis and relative protein quantification of MAMs tethering proteins IP3R1 and GRP75 in GSCs following MCU overexpression. **(G** and **H)** Representative confocal images and quantification of mitochondrial calcium levels using Rhod-2 AM (red, **G**) and ER calcium levels using Mag-Fluo-4 AM (yellow, **H**) in GSCs following MCU overexpression. **(I)** Representative confocal immunofluorescence images and quantitative colocalization analysis of Calnexin (green) and TOMM20 (red) in GSCs following OE-MCU. Data are presented as mean ± SD.

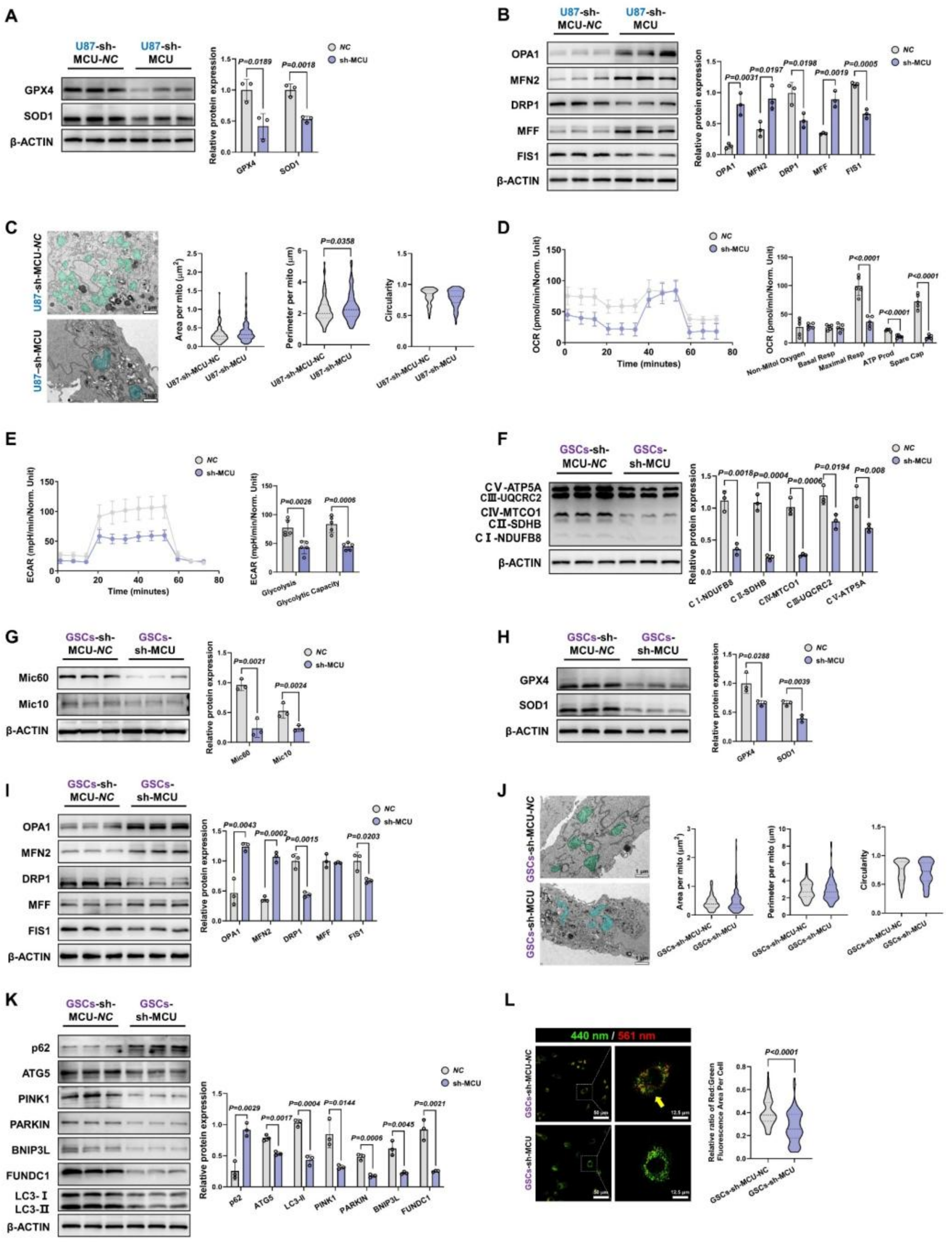


**Figure S4. MCU knockdown impairs mitochondrial dynamics, MICOS integrity, and mitophagy flux in glioma cells. (A)** Western blot analysis and relative protein quantification of antioxidant proteins (GPX4 and SOD1) in U87MG cells following MCU knockdown (sh-MCU). **(B)** Western blot analysis and relative protein quantification of mitochondrial dynamics markers (fusion proteins: OPA1, MFN2; fission proteins: DRP1, MFF, FIS1) in U87MG cells following MCU knockdown**. (C)**

Representative TEM ultrastructural images and quantitative analysis of mitochondrial morphology (area, perimeter, and circularity) in U87MG cells. (**D** and **E**) OCR (D) and ECAR (**E**) results in U87MG cells following MCU knockdown. (**F**) Western blot analysis of OXPHOS complex subunits (CI–CV) in GSCs after MCU knockdown. (**G**) Western blot analysis of Mic10 and Mic60 expression in GSCs after MCU knockdown. (**H**) Western blot analysis and relative protein quantification of GPX4 and SOD1 in GSCs following MCU knockdown. **(I**) Western blot analysis of mitochondrial dynamics markers in GSCs after MCU knockdown. (**J**) Representative TEM ultrastructural images and quantitative analysis of mitochondrial morphology in GSCs. (**K**) Western blot analysis and relative quantification of autophagy and mitophagy-related proteins (p62, ATG5, PINK1, PARKIN, BNIP3L, FUNDC1, and LC3-I/II) in GSCs after MCU knockdown. (**L**) Mito-Keima results in GSCs after MCU knockdown (excitation at 440 nm and 561 nm). Statistical analysis of the 561/440 nm ratio. Data are presented as mean ± SD.

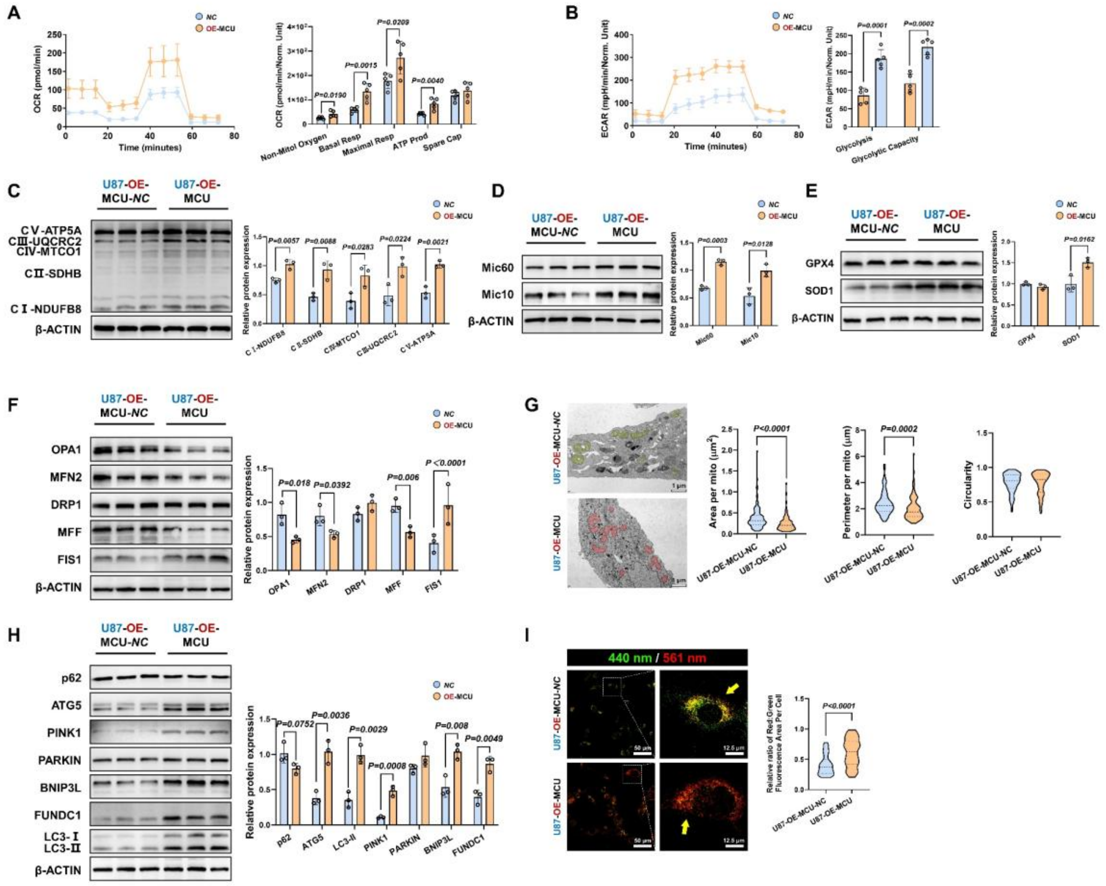


**Figure S5. Overexpression of MCU enhances mitochondrial respiration, reshapes mitochondrial dynamics, and activates the mitophagy pathway in glioma cells.** **(A)** OCR analysis evaluating mitochondrial respiration in U87MG cells following MCU overexpression (OE-MCU). **(B)** ECAR analysis assessing glycolytic function in U87MG cells following MCU overexpression. **(C)** Western blot analysis and relative protein quantification of OXPHOS complex subunits in U87MG cells following MCU overexpression. **(D)** Western blot analysis and quantification of antioxidant enzymes GPX4 and SOD1 following MCU overexpression. **(E)** Western blot analysis and relative protein quantification of the MICOS complex subunits Mic60 and Mic10 in U87MG cells following MCU overexpression. **(F)** Western blot analysis and quantification of mitochondrial fusion (OPA1, MFN2) and fission (DRP1, MFF, FIS1)

related proteins following MCU overexpression. **(G)** Representative TEM images (left) and morphometric quantification of mitochondrial area, perimeter, and circularity (right) in U87MG cells. **(H)** Western blot analysis and quantification of key autophagy and mitophagy-related proteins (p62, ATG5, LC3-I/II, PINK1, PARKIN, BNIP3L, and FUNDC1) following MCU overexpression. **(I)** Representative confocal images (left) and quantitative analysis of mitophagy flux using the Mito-Keima probe in U87MG cells. Data are presented as mean ± SD.

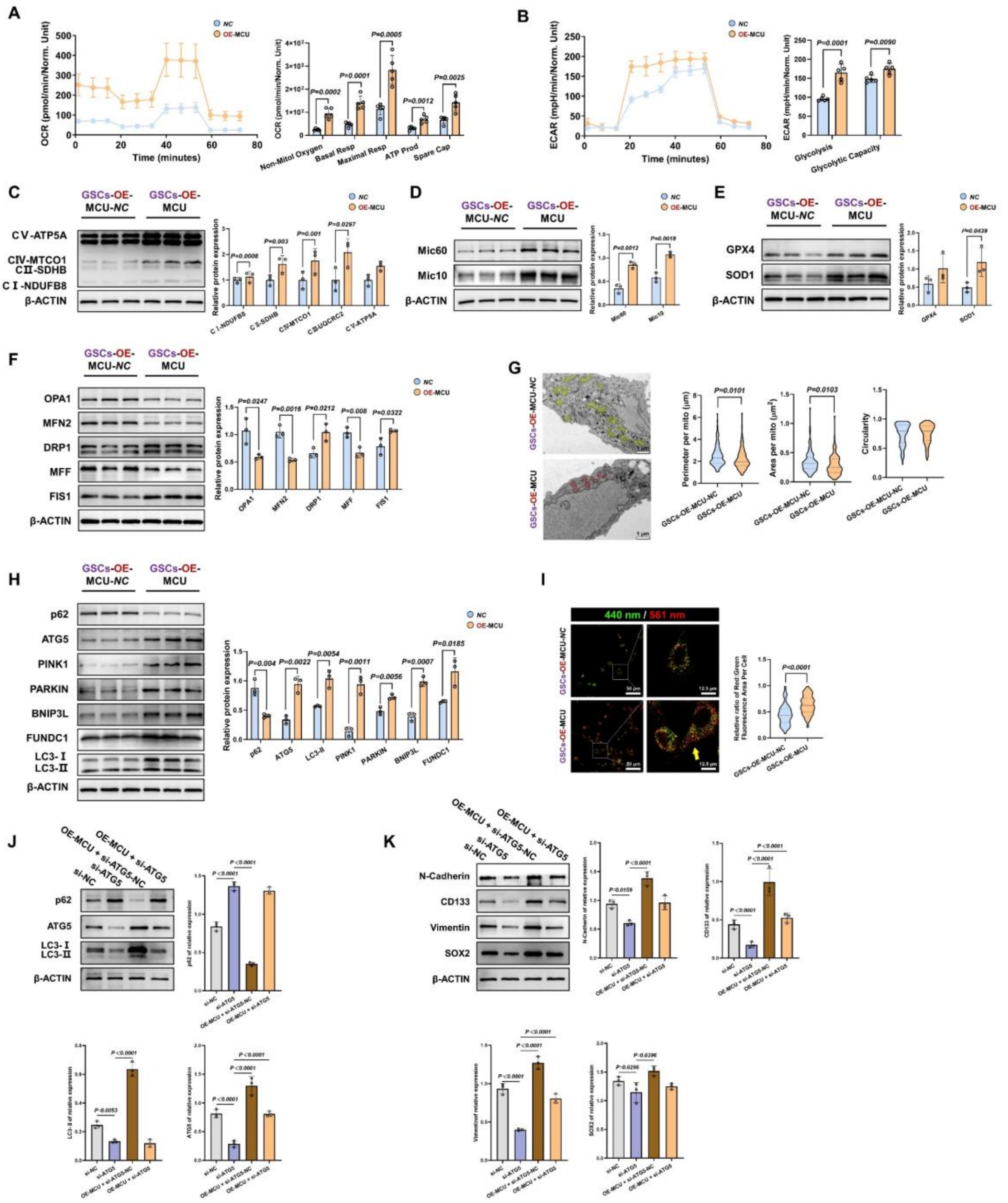


**Figure S6. Overexpression of MCU enhances mitochondrial function and promotes GSCs stemness and EMT via the mitophagy pathway. (A)** OCR analysis evaluating mitochondrial respiration in GSCs following MCU overexpression (OE-MCU). **(B)** ECAR analysis assessing glycolytic function in GSCs following MCU overexpression. **(C)** Western blot analysis and relative protein quantification of OXPHOS complex subunits (CI-CV) in GSCs following MCU overexpression. **(D)**

Western blot analysis and relative protein quantification of the MICOS complex subunits Mic60 and Mic10 in GSCs following MCU overexpression. **(E)** Western blot analysis and quantification of antioxidant enzymes GPX4 and SOD1 following MCU overexpression in GSCs. **(F)** Western blot analysis and quantification of mitochondrial fusion (OPA1, MFN2) and fission (DRP1, MFF, FIS1) related proteins following MCU overexpression. (G) Representative TEM images (left) and morphometric quantification of mitochondrial perimeter, area, and circularity (right) in GSCs. **(H)** Western blot analysis and quantification of key autophagy and mitophagy-related proteins (p62, ATG5, LC3-I/II, PINK1, PARKIN, BNIP3L, and FUNDC1) following MCU overexpression in GSCs. **(I)** Representative confocal images (left) and quantitative analysis of mitophagy flux using the Mito-Keima probe in GSCs. **(J)** Western blot analysis and quantification of autophagy markers (p62, ATG5, LC3-I/II) in GSCs following MCU overexpression and/or ATG5 knockdown (si-ATG5). **(K)** Western blot analysis and quantification of EMT markers (N-Cadherin, Vimentin) and stemness markers (CD133, SOX2) illustrating the causal rescue of MCU-driven phenotypic shifts by si-ATG5. Data are presented as mean ± SD.

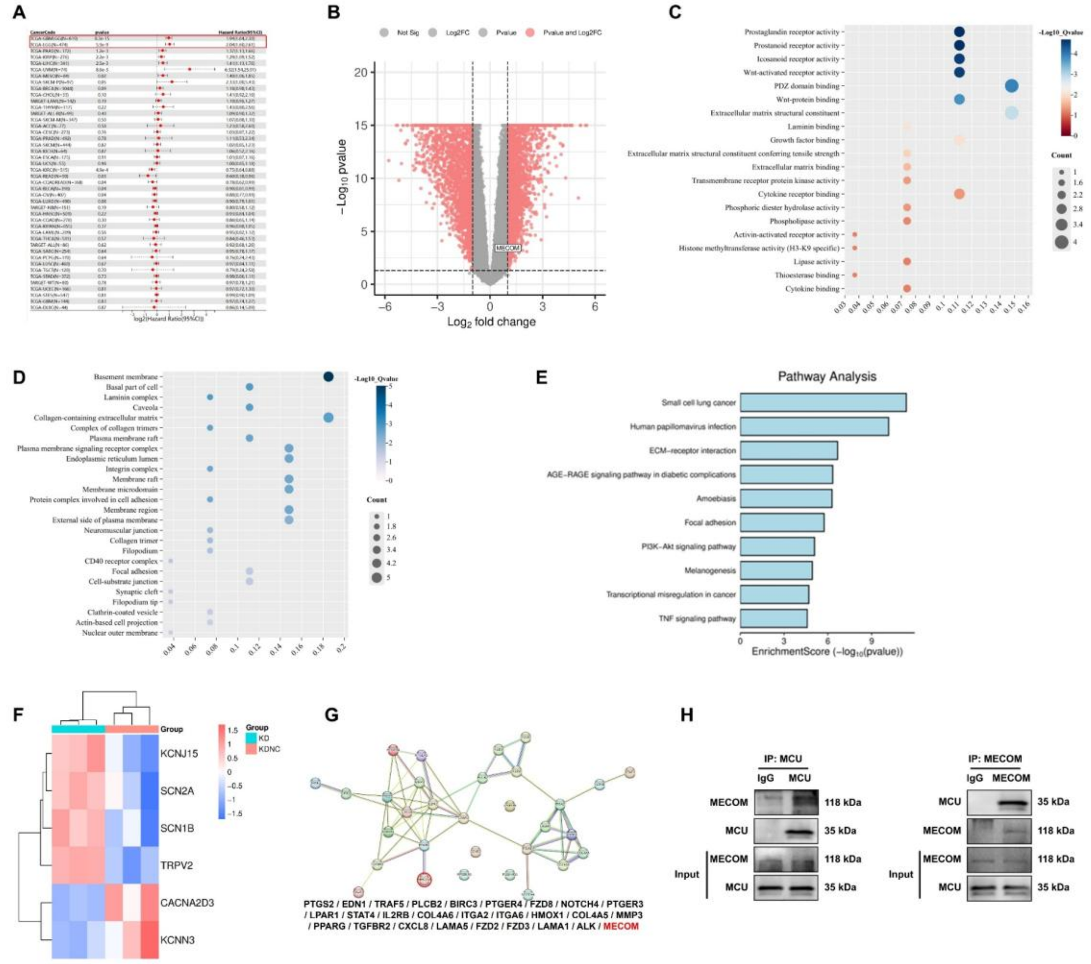


**Figure S7. Transcriptomic profiling and functional enrichment analysis revealing the MCU-MECOM regulatory network. (A)** Forest plot detailing the pan-cancer prognostic significance (hazard ratios) of MECOM across multiple TCGA cancer cohorts. The red box highlights the GBM and LGG cohorts. **(B)** Volcano plot of RNA-seq data displaying DEGs between the MCU knockdown (KD) and negative control (KDNC) groups. The significant downregulation of MECOM is explicitly highlighted. **(C and D)** Gene Ontology (GO) enrichment analysis of the identified DEGs, visualizing the top enriched terms for molecular function **(C)** and cellular component **(D)**. **(E)** KEGG pathway enrichment analysis of the DEGs, represented by

enrichment scores. **(F)** Hierarchical clustering heatmap illustrating the differential expression profiles of representative ion channel-related genes (KCNJ15, SCN2A, SCN1B, TRPV2) between the KD and KDNC groups. **(G)** PPI network of the identified cancer pathway-associated DEGs, demonstrating the topological interaction nodes of MECOM. **(H)** Co-IP assays validating the endogenous physical interaction between MCU and MECOM in GSCs.

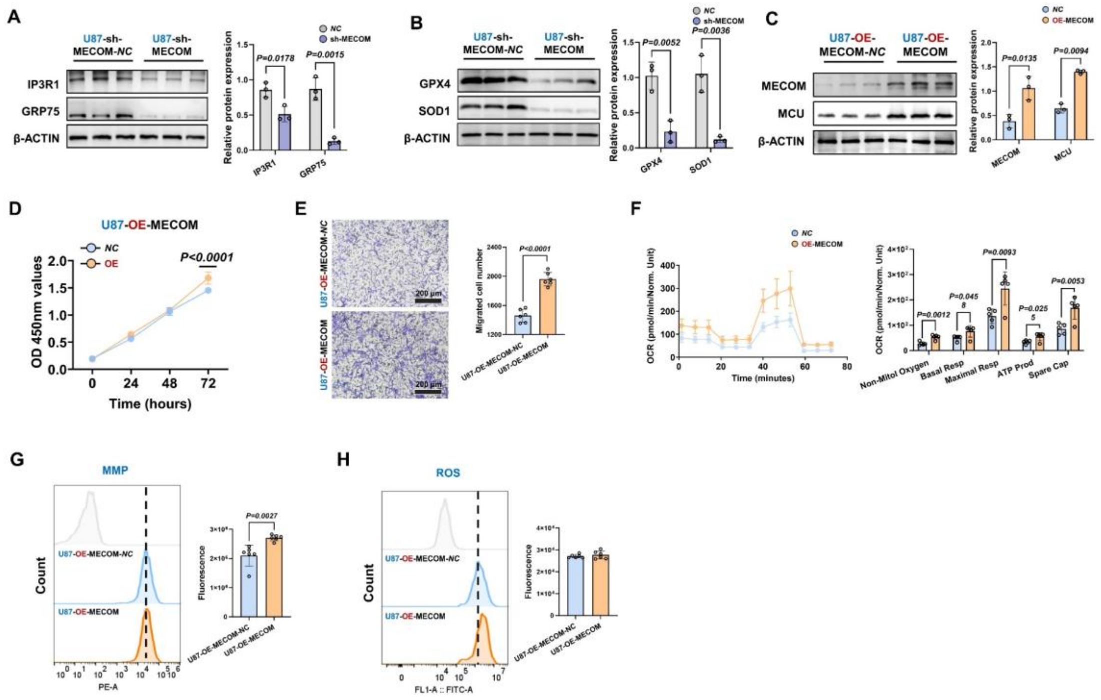


**Figure S8. MECOM regulates MAMs integrity, antioxidant capacity, and mitochondrial metabolic reprogramming in U87MG cells. (A** and **B)** WB analysis and relative protein quantification of MAMs tethering proteins (IP3R1, GRP75) **(A)** and antioxidant proteins (GPX4, SOD1) **(B)** in U87MG cells following MECOM knockdown (sh-MECOM). **(C)** WB analysis and relative protein quantification of MCU and MECOM in U87MG cells following MECOM overexpression (OE-MECOM). **(D)** CCK-8 proliferation assay of U87MG cells following MECOM overexpression over a 72-hour period. **(E)** Representative images and quantitative analysis of Transwell migration assays demonstrating increased migration in U87MG cells following MECOM overexpression. **(F)** OCR profiles and quantitative analysis of mitochondrial respiration parameters (non-mitochondrial oxygen, basal respiration, maximal respiration, ATP production, and spare capacity) in U87MG cells following MECOM overexpression. **(G** and **H)** Flow cytometry analysis and corresponding

quantification of MMP **(G)** and intracellular ROS levels **(H)** in U87MG cells following MECOM overexpression. Data are presented as mean ± SD.

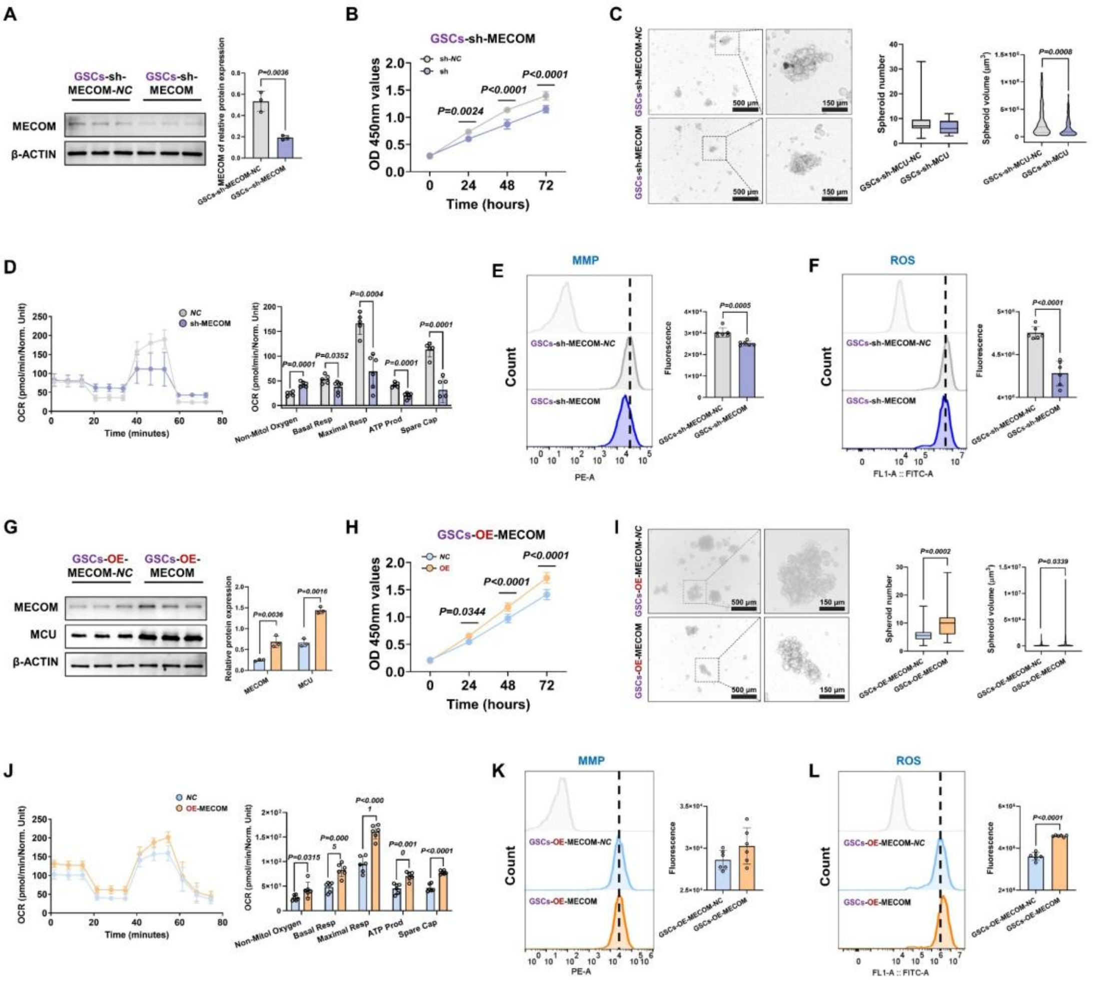


**Figure S9. MECOM regulates tumorigenicity and mitochondrial metabolic reprogramming in GSCs. (A)** WB analysis and relative protein quantification validating MECOM knockdown (sh-MECOM) in GSCs. **(B)** CCK-8 proliferation assay of GSCs following MECOM knockdown. **(C)** Representative images and statistical analysis of tumorsphere formation (spheroid number and volume) by GSCs following MECOM knockdown. **(D)** OCR profiles and quantitative analysis of mitochondrial respiration parameters in GSCs following MECOM knockdown. **(E and F)** Flow cytometry analysis and corresponding quantification of MMP **(E)** and intracellular ROS levels **(F)** in GSCs following MECOM knockdown. **(G)** WB

analysis and relative protein quantification of MECOM and MCU in GSCs following MECOM overexpression (OE-MECOM). **(H)** CCK-8 proliferation assay of GSCs following MECOM overexpression. **(I)** Representative images and statistical analysis of tumorsphere formation by GSCs following MECOM overexpression. **(J)** OCR profiles and quantitative analysis of mitochondrial respiration parameters in GSCs following MECOM overexpression. **(K** and **L)** Flow cytometry analysis and corresponding quantification of MMP **(K)** and ROS levels **(L)** in GSCs following MECOM overexpression. Data are presented as mean ± SD.

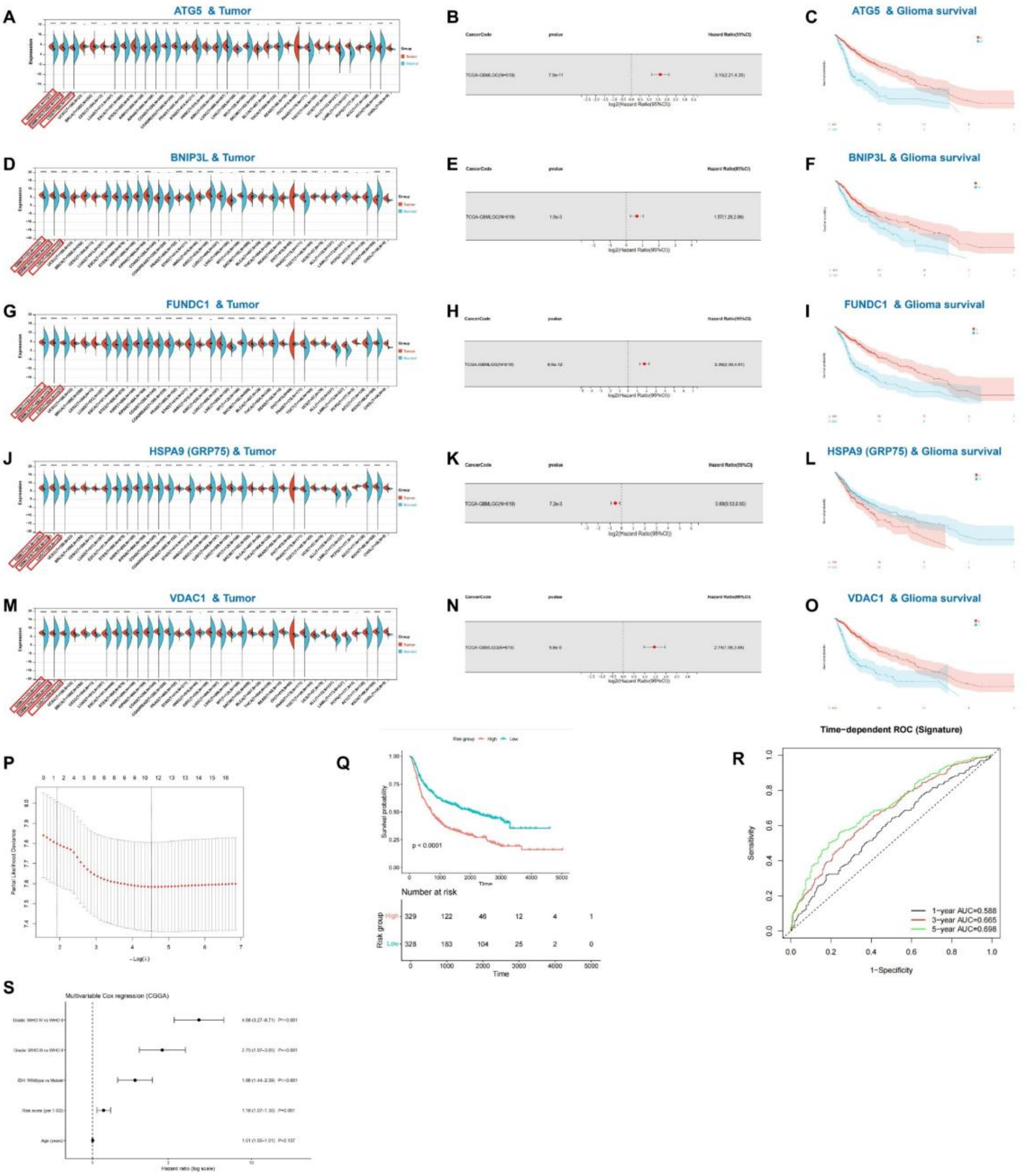


**Figure S10. Prognostic significance of individual autophagy and MAMs-associated genes and further validation of the risk signature. (A-S)** Comprehensive bioinformatic evaluation of key genes involved in mitophagy and MAMs integrity, including ATG5 **(A-C)**, BNIP3L **(D-F)**, FUNDC1 **(G-I)**, HSPA9/GRP75 **(J-L)**, and VDAC1 **(M-O)**. The left panels **(A, D, G, J, M)** display pan-cancer mRNA expression profiles comparing tumor (red) and normal (blue)

tissues, with glioma cohorts highlighted in red boxes. The middle panels **(B, E, H, K, N)** present forest plots detailing the hazard ratios (HR) of each gene within the TCGA-GBMLGG cohort. The right panels **(C, F, I, L, O)** show Kaplan-Meier overall survival (OS) curves stratifying glioma patients based on the expression levels of these respective genes. **(P)** Partial likelihood deviance plot for the selection of the optimal tuning parameter in the LASSO regression model using cross-validation. **(Q)** Kaplan-Meier OS curve demonstrating the robust prognostic discrimination of the constructed risk signature (high vs. low risk groups). **(R)** Time-dependent ROC curves evaluating the predictive accuracy of the risk signature for 1-, 3-, and 5-year overall survival. **(S)** Multivariable Cox regression forest plot in the CGGA cohort, confirming that the risk score serves as an independent prognostic indicator when adjusted for clinical features (WHO Grade, IDH mutation status, and Age).

## Supplementary tables

**Table S1. Baseline characteristics of the patients**

| Characteristics | Basic Experiments Cohort | AI Model Training & Testing Cohort | External Validation Cohort |
|---|---|---|---|
| **Total No. of patients** | 34 | 88 | 16 |
| **Sex - no. (%)** | | | |
| Male | 25 (73.5%) | 60 (68.2%) | 10 (62.5%) |
| Female | 9 (26.5%) | 28 (31.8%) | 6 (37.5%) |
| **Age - yr** | | | |
| Median | 43 | 45 | 48 |
| Range | 14 - 78 | 14 - 81 | 30 - 75 |
| **Diagnosis - no. (%)** | | | |
| Non-tumor (Cerebral hemorrhage) | 6 (17.6%) | 6 (6.8%) | 0 (0.0%) |
| Glioma (Tumor tissues) | 28 (82.4%) | 82 (93.2%) | 16 (100.0%) |
| **WHO Grade - no. (%)** | | | |
| Grade 1 | 2 (7.1%) | 6 (7.3%) | 1 (6.3%) |
| Grade 2 | 11 (39.3%) | 22 (26.8%) | 3 (18.8%) |
| Grade 3 | 5 (17.9%) | 20 (24.4%) | 4 (25.0%) |
| Grade 4 | 10 (35.7%) | 34 (41.5%) | 8 (50.0%) |
| **IDH Mutation Status - no. (%)** | | | |
| IDH1 mutant | 12 (42.9%) | 37 (45.1%) | 7 (43.8%) |
| IDH2 mutant | 1 (3.6%) | 2 (2.4%) | 0 (0.0%) |
| IDH wild-type | 13 (46.4%) | 39 (47.6%) | 8 (50.0%) |
| Missing data | 2 (7.1%) | 4 (4.9%) | 1 (6.3%) |
| **Tumor Classification - no. (%)** | | | |
| Pilocytic Astrocytoma | 2 (7.1%) | 6 (7.3%) | 1 (6.3%) |
| Astrocytoma, IDH-mutant | 5 (17.9%) | 15 (18.3%) | 3 (18.8%) |
| Oligodendroglioma, IDH-mutant and 1p/19q co-deleted | 6 (21.4%) | 22 (26.8%) | 4 (25.0%) |

| Characteristics | Basic Experiments Cohort | AI Model Training & Testing Cohort | External Validation Cohort |
| --- | --- | --- | --- |
| Glioblastoma, IDH-wild type | 8 (28.6%) | 32 (39.0%) | 8 (50.0%) |
| Missing data | 7 (25.0%) | 7 (8.5%) | 0 (0.0%) |

## Table S2. Lentivirus used in the study

| Lentivirus | Groups | Vendor | Item No. | Lentivirus vector | Titer (TU/mL) |
|---|---|---|---|---|---|
| MCU | sh-MCU-NC | Hanbio | HH20231101ZY-LV01 | HBLV-LUC-PURO NC | 1.5*10^8 |
| MCU | sh-MCU | Hanbio | HH20231101ZY-LV01 | HBLV-h-MCU shRNA1-LUC-PURO | 2*10^8 |
| MCU | OE-MCU-NC | Hanbio | HH20231101ZY-LV02 | HBLV-LUC-PURO | 3.0*10^8 |
| MCU | OE-MCU | Hanbio | HH20231101ZY-LV02 | HBLV-h-MCU-3xflag-LUC-PURO | 3*10^8 |
| MECOM | sh-MECOM-NC | Hanbio | HH20240719ZY-LV01 | HBLV-LUC-PURO NC | 1.5*10^8 |
| MECOM | sh-MECOM | Hanbio | HH20240719ZY-LV01 | HBLV-h-MECOM shRNA2-LUC-PURO | 2*10^8 |
| MECOM | OE-MECOM-NC | Hanbio | HH20240719ZY-LV02 | HBLV-LUC-PURO | 3.0*10^8 |
| MECOM | OE-MECOM | Hanbio | HH20240719ZY-LV02 | HBLV-h-MECOM-3xflag-LUC-PURO | 4*10^8 |
| Mito-Keima | - | Hanbio | HH20240621ZY-LV01 | | 3.16*10^10 |

**Table S3. Antibodies used for WB / IF / IHC**

| Antibody | Source | Vendor | Dilution | Cat. # | WB / IF / IHC |
|---|---|---|---|---|---|
| β-Actin | Rabbit | Servicebio | 1:3000 | GB15003 | WB |
| DRP1 | Rabbit | Cell signaling Technology | 1:1000 | 8570 | WB |
| p-DRP1 Ser616 | Rabbit | Invitrogen | 1:1000 | PA5-100917 | WB |
| MCU | Rabbit | Abcam | 1:1000 | ab272488 | WB / IHC / Co-IP |
| MCU | Rabbit | Abcam | 1:200 | ab219827 | IF |
| VDAC1 | Rabbit | ABclonal | 1:1000 | A19707 | WB |
| IP3R1 | Rabbit | ABclonal | 1:1000 | A4436 | WB |
| GRP75 | Rabbit | ABclonal | 1:1000 | A11256 | WB |
| OPA1 | Mouse | Santa | 1:1000 | sc-393296 | WB |
| MFF | Rabbit | Cell signaling Technology | 1:1000 | 84580 | WB |
| MFN2 | Rabbit | Cell signaling Technology | 1:1000 | 9482 | WB |
| PARKIN | Rabbit | Invitrogen | 1:1000 | PA5-13399 | WB |
| PINK1 | Rabbit | Invitrogen | 1:1000 | PA5-86941 | WB |
| SOD1 | Rabbit | ABclonal | 1:1000 | A0274 | WB |
| GPX4 | Rabbit | ABclonal | 1:1000 | A25009 | WB |
| ATG5 | Rabbit | ABclonal | 1:1000 | A19677 | WB |
| CD133 | Rabbit | Servicebio | 1:800 | GB114579-100 | IF |
| FUNDC1 | Rabbit | ABclonal | 1:1000 | A16318 | WB |
| Ki-67 | Rabbit | Servicebio | 1:500 | GB151142-100 | IF |
| LC3B | Rabbit | Abcam | 1:1000 | ab192890 | WB |
| BNIP3L | Rabbit | ABclonal | 1:1000 | A6283 | WB |
| p62 | Rabbit | ABclonal | 1:1000 | A19700 | WB |
| SOX2 | Rabbit | Servicebio | 1:500 | GB11249-100 | IF |

| **Antibody** | **Source** | **Vendor** | **Dilution** | **Cat. #** | **WB / IF / IHC** |
|---|---|---|---|---|---|
| Mic10 | Rabbit | Proteintech | 1:1000 | 12494-1-AP | WB |
| Mic60 | Rabbit | Proteintech | 1:1000 | 10179-1-AP | WB |
| Total OXPHOS Rodent WB Antibody Cocktail | Rabbit | Abcam | 1:1000 | ab110413 | WB |
| Calnexin | Rabbit | Abcam | 1:1000 | ab22595 | WB |
| COX IV | Rabbit | Abcam | 1:1000 | ab202554 | WB |
| MECOM | Rabbit | Invitrogen | 1:1000 | MA5-14892 | WB / IF / IHC / Co-IP |
| HRP | Mouse | Proteintech | 1:5000 | RGAM001 | WB |
| TOMM20 | Rabbit | Invitrogen | 1:1000 | PA5-52843 | WB / IF |
| N-Cadherin | Rabbit | Invitrogen | 1:1000 | PA5-29570 | WB |
| Vimentin | Rabbit | Invitrogen | 1:1000 | MA5-11883 | WB |
| HRP | Rabbit | Proteintech | 1:5000 | RGAR001 | WB |
| HRP | Rabbit | Abbkine | 1:1000 | A21020 | IF |

**Table S4. Fluorescent probes and key chemical reagents**

| Probe / Reagent / Kit | Vendor | Cat. # |
|---|---|---|
| **Fluorescent Probes & Indicators** | | |
| Rhod-2 AM | MedChemExpress | HY-101896 |
| Mag-Fluo-4 AM | MedChemExpress | HY-D1498 |
| CellROX® Deep Red reagent | Thermo Fisher Scientific | C10491 |
| MitoSOX™ Red | Thermo Fisher Scientific | M36008 |
| JC-1 Dye (200×) | Beyotime | C2006-1 |
| TMRE Staining Solution | Beyotime | C2001S |
| DCFH-DA | Beyotime | S0033S-1 |
| **Pharmacological Modulators & Controls** | | |
| MCU-i11 | MedChemExpress | HY-119106 |
| Spermine | MedChemExpress | HY-B1777 |
| Chloroquine (CQ) | Thermo Fisher Scientific | J64459 |
| CCCP | Beyotime | C2006-4 |
| ROS Inducer | Beyotime | S0033S-2 |
| Recombinant human bFGF | MedChemExpress | HY-P7004 |
| Recombinant human EGF | MedChemExpress | HY-P7109 |
| **Functional Assay Kits & Transfection** | | |
| Cell Mitochondria Isolation Kit | Beyotime | C3601 |
| Seahorse XF Cell Mito Stress Test Kit | Agilent | 103015-100 |
| Seahorse XF Glycolysis Stress Test Kit | Agilent | 103020-100 |
| Cell Counting Kit-8 (CCK-8) | Solarbio | CA1210-500T |
| Lipofectamine™ 3000 | Thermo Fisher Scientific | L3000001 |

**Table S5. Information of siRNA transfection**

| Target gene | Species | Modulation type | Reagent format | RefSeq ID | Catalog No. | Supplier |
|---|---|---|---|---|---|---|
| ATG5 | Human | Knockdown | siRNA | NW_027138159 | RX0150718 | Kereis |
| Control | N/A | Negative Control | Scrambled siRNA | - | RX0150719 | Kereis |
